# Differential Loss of OAS Genes Indicates Diversification of Antiviral Immunity in Mammals

**DOI:** 10.3390/vaccines11020419

**Published:** 2023-02-12

**Authors:** Leopold Eckhart, Wolfgang Sipos

**Affiliations:** 1 Department of Dermatology, Medical University of Vienna, 1090 Vienna, Austria; 2 Clinical Department for Farm Animals and Herd Management, University of Veterinary Medicine Vienna, 1210 Vienna, Austria

**Keywords:** oligoadenylate synthetase, innate immunity, evolution, zoonoses, SARS-CoV-2, camel, bat, gene loss, gene duplication, gene family

## Abstract

One of the main mechanisms of inducing an antiviral response depends on 2′-5′-oligoadenylate synthetases (OAS), which sense double-stranded RNA in the cytoplasm and activate RNase L. Mutations leading to the loss of functional *OAS1* and *OAS2* genes have been identified as important modifiers of the human immune response against severe acute respiratory syndrome coronavirus 2 (SARS-CoV-2). Here, we performed comparative genomics to search for inactivating mutations of *OAS* genes in other species of mammals and to establish a model for the diversifying evolution of the *OAS* gene family. We found that a recombination of the *OAS* and *OAS-like* (*OASL*) loci has led to the loss of *OAS2* in camelids, which also lack *OAS3*. Both paralogs of *OASL* and *OAS3* are absent in Asian pangolins. An evolutionarily ancient *OAS* paralog, which we tentatively name *OAS4*, has been lost in pangolins, bats and humans. A previously unknown *OAS* gene, tentatively named *OAS5*, is present in Yangochiroptera, a suborder of bats. These differences in the *OAS* gene repertoire may affect innate immune responses to coronaviruses and other RNA viruses.

## 1. Introduction

**Figure 1:**
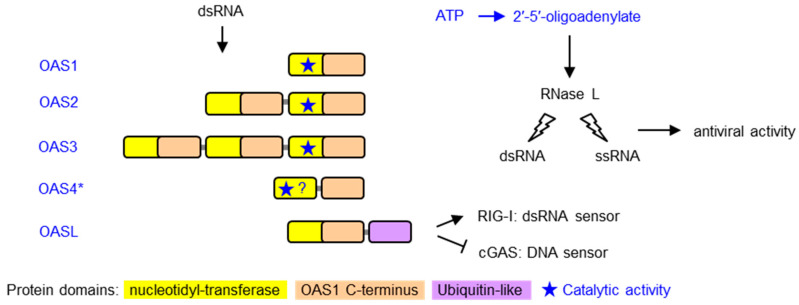
Domain organization and mechanism of antiviral activity of OAS family proteins. Proteins of the 2′-5′-oligoadenylate synthetase (OAS) family are depicted schematically. Domains are color-coded as described at the bottom of the figure. OAS proteins are characterized by the presence of one or more nucleotidyl-transferase (cd05400: NT_2-5OAS_ClassI-CCAase) and OAS1 C-terminal (pfam10421: OAS1_*C*) [8] domains. The location of the catalytically active site is indicated by a star. OASL proteins contain a ubiquitin-like domain at the carboxy-terminus and lack catalytic activity. Upon binding to double-stranded RNA (dsRNA), OAS proteins catalyze the oligomerization of ATP to 2′-5′-oligoadenylate, which activates RNase L and thereby induces the degradation of dsRNA and single-stranded RNA (ssRNA) to suppress virus replication. OASL activates RIG-I, a sensor of cytoplasmic dsRNA, and suppresses cGAS, a sensor of cytoplasmic DNA *, OAS4 is the tentative name of an OAS paralog that was identified by Wang and colleagues who described the corresponding gene as “*OAS1* in the *ERAP2* (endoplasmic reticulum amino peptidase 2)-*RIOK2* (RIO kinase 2) region (E-R region)” [9].

Mammalian cells have different mechanisms to sense viral infections and initiate an innate immune response. One of the main approaches is the binding of specific sensor proteins to nucleic acids with infection-associated features, such as cytoplasmic localization of DNA, presence of double-stranded RNA or presence of Z-nucleic acid structures [1,2,3]. These sensors trigger signaling cascades that lead to reactions of the infected cell, the tissue and the immune system aimed at stopping the replication and spread of viruses. 2′-5′ oligoadenylate synthetases (OASs) bind double-stranded (ds) RNA in the cytoplasm and subsequently catalyze the oligomerization of ATP to 2′-5′-oligoadenylate. This oligomer activates RNase L, which subsequently degrades viral RNA to suppress the replication of the virus [1]. RNA degradation also generates small self-RNA, which amplifies antiviral innate immunity by binding to other receptors [4]. Humans have three OAS proteins comprising either one, two or three repeats of a nucleotidyl-transferase (NT) and an OAS1 C-terminal (OAS1C) domain. Besides catalytically active OAS enzymes, an OAS-like (OASL) protein contributes to the control of signaling in response to dsRNA in the cytoplasm. OASL activates retinoic acid inducible gene I (RIG-I), a sensor of cytoplasmic dsRNA [5,6], and suppresses cyclic GMP-AMP synthase (cGAS), a sensor of cytoplasmic DNA, leading to reduced replication of RNA viruses and enhanced replication of DNA viruses [7] (Figure 1).

The OAS-RNase L pathway is involved in the defense against many viruses, some of which have evolved counteractive strategies [10,11,12,13]. Recent research has demonstrated that this pathway is also involved in the human immune response against severe acute respiratory syndrome coronavirus 2 (SARS-CoV-2) [14]. Specifically, mutations inactivating OAS1, OAS2 or RNase L impair the normal immune response and lead to SARS-CoV-2–related multisystem inflammatory syndrome in children [14]. OAS1 was reported to inhibit SARS-CoV-2 through its prenylated isoform [15]. Interestingly, the antiviral activity of OAS1 differs among primates due to mutations of its amino acid sequence, suggesting that a reduction in or loss of OAS1 catalytic activity may have had advantages in evolution [16]. Another report linked the decay of *OAS1* mRNAs with the risk of COVID-19 hospitalization [17].

*OAS* genes are evolutionarily ancient [9,18]. Species from the major phylogenetic metazoan clades contain different sets of *OAS* genes, and even within tetrapods, significant variation has been reported [9,19]. For instance, *OAS1* has undergone duplications in rodents and cattle [15,20]. In contrast to humans, some other placental mammals and marsupials have two copies of *OASL* [18], indicating that two *OASL* genes were also present in the genome of evolutionary ancestors of humans, and one of these copies was lost in the lineage leading to humans. Recently, a new *OAS* paralog, hereafter referred to as *OAS4*, was reported to be present in some amphibian, sauropsid and mammalian species [9]. Phylogenetic analysis suggested that this paralog emerged earlier in evolution than *OAS1*, *OAS2* and *OAS3* and that it was lost in rodents and primates [9]. Likewise, *OAS3* has been lost in cetartiodactyls and in the tree shrew [20,21].

Comparative genomic studies have revealed a high diversity of innate immune genes in mammals with striking cases of gene degeneration in bats and pangolins [22,23,24,25,26]. Genes involved in the sensing of cytoplasmic DNA, such as *CGAS* and *STING1*, and cytoplasmic RNA, such as *IFIH1*/MDA5 and *ZBP1*, have been lost during the evolution of pangolins [23,24]. As bats are considered a likely source of SARS-CoV-2 and pangolins possibly were intermediate hosts of this virus [27,28], we put forward the hypothesis that alterations in antiviral innate immunity may contribute to the differential persistence of viruses in populations of such species with the risk of virus spillover potentially causing pandemics.

Here, we extended the concept of gene loss as a driver of inter-species variation in innate immunity and screened a selected group of mammalian species for cases of gene loss in the *OAS* gene family. The results of this study have implications for comparative immunology, and the selection of animal models for studying host–virus interactions.

## 2. Materials and Methods

Comparative genomics was performed according to an approach reported previously [23,29]. Nucleotide and amino acid sequences were downloaded from GenBank. Accession numbers are indicated in the text. Genes were identified in the genome sequences of *Homo sapiens*, assembly: GRCh38.p14 (GCF_000001405.40); *Camelus bactrianus*, assembly: Ca_bactrianus_MBC_1.0 (GCF_000767855.1); *Camelus dromedarius*, assembly: CamDro3 (GCF_000803125.2); *Vicugna pacos*, assembly: VicPac3.1 (GCF_000164845.3); *Canis familiaris*, assembly: ROS_Cfam_1.0 (GCF_014441545.1); *Manis javanica*, assembly: YNU_ManJav_2.0 (GCF_014570535.1); *Manis pentadactyla*, assembly: YNU_ManPten_2.0 (GCF_014570555.1); *Rhinolophus sinicus*, assembly: ASM188883v1 (GCF_001888835.1); *Molossus molossus*, assembly: mMolMol1.p (GCF_014108415.1); *Myotis myotis*, assembly: mMyoMyo1.p (GCF_014108235.1); and *Artibeus jamaicensis*, assembly: WHU_Ajam_v2 (GCF_014825515.1).

The Basic Local Alignment Search Tool (BLAST) [30] was used to determine sequence similarities. Sequence alignments were made with Multalin (http://multalin.toulouse.inra.fr/multalin/, accessed on 30 December 2022) and MUSCLE (https://www.ebi.ac.uk/Tools/msa/muscle/, accessed on 30 December 2022). Protein domains were identified with the NCBI Conserved Domain search tool [31]. Phylogenetic relationships and divergence times of phylogenetic lineages were obtained from the Timetree website (www.timetree.org, accessed on 30 December 2022) [32].

## 3. Results

### 3.1. Comparative Genomics Reveals Loss of OAS Genes during the Evolution of Humans and Camelids

We performed comparative genomics to determine the presence or absence of *OAS* genes in a selected subset of mammalian species. This study was focused on humans and clades of mammals (camelids, pangolins and bats), which were suspected or confirmed as reservoirs of coronaviruses with zoonotic potential [27,33,34,35]. An overview of the distribution of *OAS* family genes in the various species is provided in Table 1. The GenBank accession numbers of proteins encoded by these genes are listed in Table S1, and the corresponding amino acid sequences are documented in Figure S1. Together with the knowledge of phylogenetic relationships of mammals [32], the distribution of *OAS* paralogs in the various species allowed us to infer which genes were lost or gained during the evolutionary history of particular species or clades.

**Figure 2:**
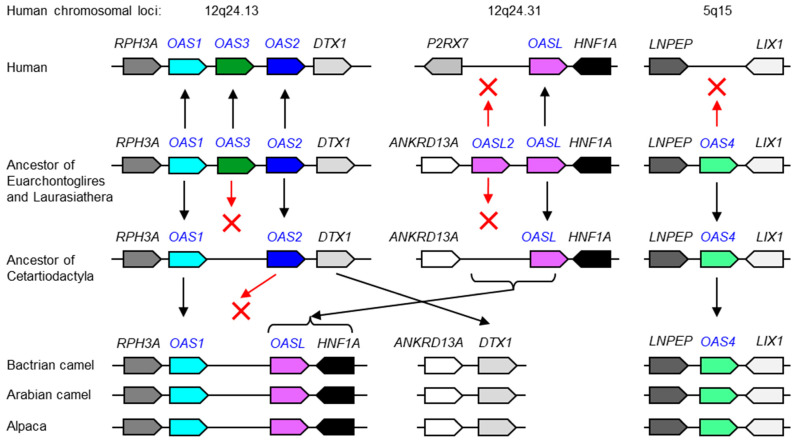
Evolution of *OAS* gene loci in camelids in comparison to humans based on comparative genomics. Gene loci are schematically depicted. Genes are represented by rightwards and leftwards finger-post arrow symbols pointing in the direction of transcription. Note that, for simplification, genes that are not conserved among species have been omitted. The arrangement of genes in ancestors is inferred from shared patterns of gene arrangement (synteny) in extant species. Inheritance of genes is indicated by upwards and downwards arrows. Red Xs indicate gene loss. The scientific names of the species and accession numbers of proteins encoded by *OAS* family genes are provided in Table S1. Chromosomal loci of the human *OAS* gene family are shown at the top of the figure.

The gene loci of the *OAS* family share the same neighboring genes (synteny) in many but not all species. Comparative analysis indicated that chromosomal positions 12q24.13, 12q24.31 and 5q15 in the human genome correspond to the evolutionarily ancestral loci of *OAS* gene paralogs. The conservation of *OAS4* in cetartiodactyls (Figure 2), African elephant and mouse lemur (Figure S1), and the absence of an *OAS4* gene at human chromosome 5q15 indicated that, in agreement with a recent report [9], *OAS4* was lost in the human lineage (Figure 2). Likewise, one of two ancestral *OASL* genes was lost during the evolution of humans (Figure 2).

Camelids, which are a subclade of cetartiodactyls, have lost *OASL2* but retained *OAS4* (Figure 2). In line with a previous report on artiodactyls [20], *OAS3* is absent in camels and alpaca (Figure 2). Camelids are unique within terrestrial cetartiodactyls because they lack *OAS2* (Figure 2). The unusual tandem arrangement of *OAS1* and *OASL* in camels and alpacas indicates that a recombination event occurred in a common ancestor of camelids, and *OAS2* was probably lost in the course of this chromosomal rearrangement.

### 3.2. Pangolins have Lost Multiple OAS Genes

**Figure 3:**
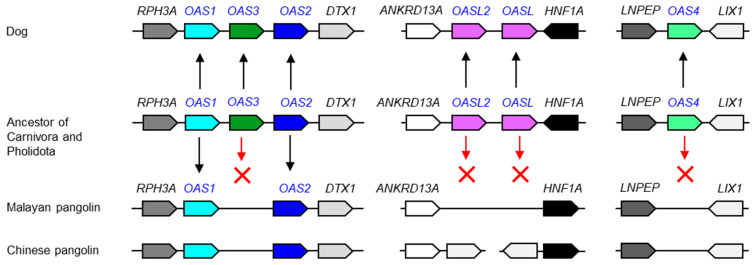
Evolution of *OAS* gene loci in the dog and the pangolin. Gene loci of the *OAS* family are schematically depicted with symbols described in the legend of Figure 2. The scientific names of the species and accession numbers of proteins encoded by *OAS* family genes are provided in Table S1.

Next, we investigated pangolins, which are carriers of SARS-CoV-2-like viruses [28] and have a degenerated set of innate immune genes [23,24,25]. Pangolins constitute the clade Pholidota, which is most closely related to Carnivora (dog-like and cat-like mammals). Therefore, we compared the genomes of the Malayan pangolin (*Manis javanica*), Chinese pangolin (*Manis pentadactyla*) and the dog. In contrast to the dog, which has the full set of ancestral *OAS* genes, the Asian pangolins lack *OAS3*, *OAS4*, and both *OASL1* and *OASL2* (Figure 3 and Figure S2). At present, gene annotations are not available for African pangolins. Due to the loss of four ancestral genes, the *OAS* gene family is massively degenerated in Malayan and Chinese pangolins (Figure 3).

### 3.3. The Evolution of Bats Was Associated with the Diversification of OAS Paralogs

**Figure 4:**
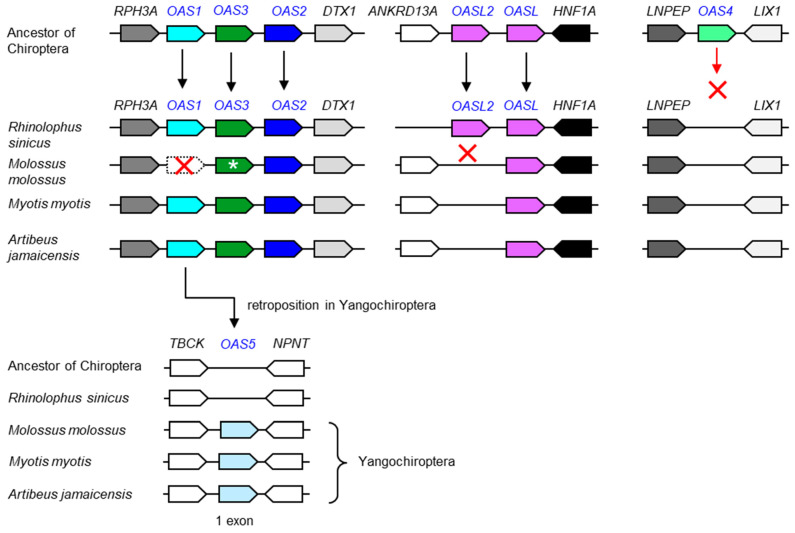
Evolution of *OAS* gene loci in bats. Gene loci of the *OAS* family are schematically depicted with symbols described in the legend of Figure 2. For simplification, genes that are not conserved among species have been omitted. The arrangement of genes in ancestors is inferred from shared patterns of gene arrangement (synteny) in extant species. The open reading frame *OAS1* of *Molossus molossus* is disrupted by point mutations. *OAS3* of *Molossus molossus* (green symbol with a white asterisk) contains more exons than its orthologs in other species and is predicted to encode a protein with 6 repeats of the NT and OAS1C domains (Figure S3). The gene tentatively named *OAS5* consists of only one exon, suggesting that it originated by retroposition [41]. GenBank accession numbers of *OAS5* genes: LOC118635736 (*Molossus molossus*), LOC118673356 (*Myotis myotis*), LOC119056133 (*Artibeus jamaicensis*). Species: Chinese rufous horseshoe bat (*Rhinolophus sinicus*), Pallas’s mastiff bat (*Molossus molossus*), greater mouse-eared bat (*Myotis myotis*), Jamaican fruit bat (*Artibeus jamaicensis*). Accession numbers of proteins encoded by *OAS* family genes are provided in Table S1.

**Figure 5:**
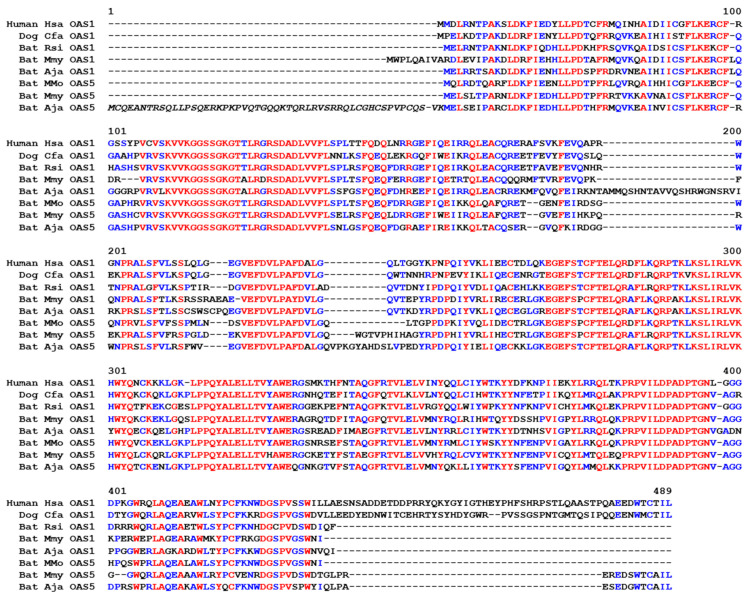
Amino acid sequence alignment of OAS1 and OAS5 proteins. Amino acid sequences were aligned with the Multalin program. Red fonts indicate residues conserved in all sequences; blue fonts indicate residues conserved in at least 50% of sequences. The italicized segment of the sequence Bat-Aj_OAS5 is likely to correspond to an erroneous extension of the coding sequence in the protein prediction of GenBank. The accession numbers of the sequences are shown in Figure S1. Numbers above the sequences indicate amino acid sequence positions. Species: Hsa, *Homo sapiens*; Cfa, *Canis familiaris*; Rsi, *Rhinolophus sinicus*; Mmo, *Molossus molossus*; Mmy, *Myotis myotis*; Aja, *Artibeus jamaicensis*.

Bats, comprising the phylogenetic clade Chiroptera, have special adaptations of the immune system that allow them to act as reservoirs of many viruses [36,37,38,39], most likely including the virus from which SARS-CoV-2 evolved [27]. We performed an exploratory analysis of *OAS* genes in a subset of bats and found that *OAS2*, *OAS3* and at least one *OASL* gene are conserved in bats (Figure 4). *OAS1* is also present in all species investigated; however, the *OAS1* ortholog of *Molossus molossus* contains inactivating mutations. OAS3 of the same species is predicted to contain an extended number of domains (Figure S3). The sequence modifications and their impact on the gene function remain to be investigated because sequence confirmations and analyses of gene transcripts were not within the scope of the present study. *OAS4* was absent in all bats investigated, suggesting that this gene has been lost (Figure 4). Unexpectedly, we identified an as-yet-uncharacterized paralog, tentatively named *OAS5*, by sequence similarity searches in the genomes of species of the suborder Yangochiroptera [40]. *OAS5* is located between the *TBCK* and *NPNT* genes, a locus that does not contain an *OAS* paralog in any species investigated except those of the clade Yangochiroptera. *OAS5* genes encode proteins with high sequence similarity to OAS1 (Figure 5). In contrast to all other known *OAS* paralogs, *OAS5* does not have introns, so the open reading frame is entirely contained in a single exon, suggesting that *OAS5* has arisen by reverse transcription of an *OAS1* mRNA followed by insertion of the complementary DNA into the genome of a germ cell in an ancestor of Yangochiroptera (Figure 4).

**Figure 6:**
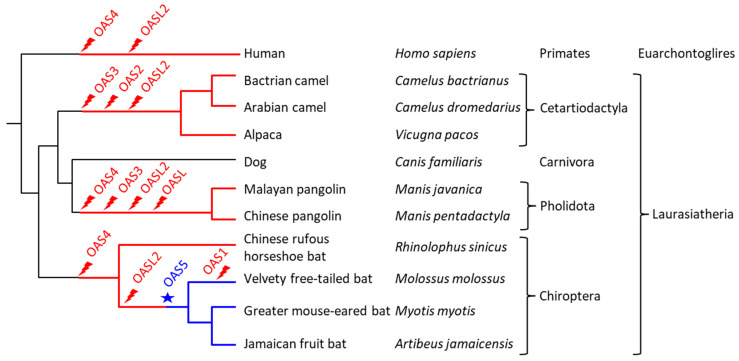
Gene loss and gain events mapped onto a phylogenetic tree of the species investigated in this study. The tree on the left shows the relationship between species. Higher taxonomic ranks are shown on the right. Gene loss (flash symbol, red) and gain (star, blue) are mapped onto this tree on the basis of gene absence or presence in the extant species. Red lines indicate lineages in which at least one member of the *OAS* gene family was missing. Blue lines indicate the presence of *OAS5* in Yangochiroptera. The chronological order of gene loss events between nodes of the tree is not known.

Taken together, these data show that the *OAS* gene family underwent lineage-specific changes leading to significant differences in the repertoire of *OAS* paralogs in major clades of extant mammals (Figure 6).

## 4. Discussion

The OAS–RNase L pathway is one of the central mechanisms of antiviral defense. A large body of literature exists on the roles of *OAS* genes in human cells and cells of model organisms [2,42,43,44]. It was noted early on that the *OAS* gene complement differs between humans and rodents as the latter have an amplification of *OAS1* genes [45]. Studies of other species, including many domestic and wild mammals, revealed further inter-species differences [18,20,21,46]. The present study extends the comparative analysis of the *OAS* family to a set of species implicated in the spread of coronaviruses and reveals previously unknown taxon-specific compositions of the *OAS* gene family. 

Our results show that the *OAS* gene family is larger than the set of *OAS1*, *OAS2*, *OAS3* and *OASL* genes in humans. Two copies of *OASL* genes have previously been identified in other species, and an as-yet-uncharacterized OAS paralog, which we name *OAS4*, was reported previously [9]. The present study identified another paralog, tentatively named *OAS5*, in a subgroup of bats. It will be important to investigate OAS4 and OAS5 proteins with regard to RNA-binding properties, catalytic activities and functions in antiviral defense and other processes.

Importantly, peculiar features of the *OAS* gene sets were found in camelids, pangolins and bats, which have been implicated in the origin of viral zoonoses [27,33,34]. These findings provide a basis for studying the impact of particular *OAS* gene combinations on the induction of anti-viral defense in follow-up studies. For a comprehensive evaluation of the significance of *OAS* genes in zoonoses, reports on other hosts of coronaviruses [27,47,48] and zoonoses involving other viruses should also be considered [35,49,50,51,52].

Vaccines play key roles in the fight against viral pathogens in human and veterinary medicine. Therefore, it is of special clinical interest to transfer basic immunological knowledge into the development of highly effective vaccines. OAS proteins are involved in early immune responses against RNA viruses and perhaps also against modified-live RNA virus vaccines [10,53]. Yellow fever vaccination induced upregulation of *OAS1* among other innate antiviral molecules and also a strong acquired serological and cellular immune response [54]. A drawback in the development of vaccines directed against RNA viruses is their generally high rate of sequence mutations, as exemplified by one of the economically most important RNA viruses in veterinary medicine, the porcine reproductive and respiratory syndrome virus (PRRSV) [55,56]. Despite its high mutation rate, it is possible to design effective vaccines [57]. The PRRSV is an ssRNA virus, but dsRNA intermediates are formed during intracellular replication. Thus, veterinary species such as pigs could serve as models for investigating the role of *OAS* gene family members and other components of innate immunity in modulating the efficacy of vaccines.

This study has limitations that need to be considered in the interpretation of the data. First, most genes were predicted by automated computational analysis, which is used by GenBank to annotate genes of non-model species [58]. Although these predictions are useful for compiling orthologous genes, structural details of the predicted genes need to be corrected in some cases [59,60]. Second, the structure of the predicted mRNAs and proteins has not been experimentally confirmed yet. However, the mapping of RNA-seq reads onto the GenBank genome sequences confirms that the predicted exons are indeed present in mature mRNAs of many species investigated (Figure S3A). Finally, the proteins encoded by the predicted *OAS* family genes remain to be characterized with regard to their biochemical features and their roles in intracellular signaling in response to viral infections.

The differences in the conservation of established *OAS* family members, *OAS1*, *OAS2*, *OAS3*, *OASL* and *OASL2*, and possibly also the presence or absence of *OAS4* and *OAS5,* may contribute to differences in responses to viral dsRNA in mammalian species. Studies are warranted to determine the impact of specific *OAS* gene sets on the control of viruses in phylogenetically diverse mammals [39,49,61,62].

## Figures and Tables

**Table 1:** Conservation of *OAS* genes in mammalian species investigated in this study.

Species	Binomial Name	*OAS1*	*OAS2*	*OAS3*	*OAS4*	*OAS5*	*OASL*	*OASL2*
Human	*Homo sapiens*	+	+	+	–	–	+	–
Bactrian camel	*Camelus bactrianus*	+	–	–	+	–	+	–
Arabian camel	*Camelus dromedarius*	+	–	–	+	–	+	–
Alpaca	*Vicugna pacos*	+	–	–	+	–	+	–
Dog	*Canis familiaris*	+	+	+	+	–	+	+
Malayan pangolin	*Manis javanica*	+ *	+	–	–	–	–	–
Chinese pangolin	*Manis pentadactyla*	+	+	–	–	–	–	–
Chinese rufous horseshoe bat	*Rhinolophus sinicus*	+	+	+	–	–	+	+
Pallas’s mastiff bat	*Molossus molossus*	+ *	+	+	–	+	+	–
Greater mouse-eared bat	*Myotis myotis*	+	+	+	–	+	+	–
Jamaican fruit bat	*Artibeus jamaicensis*	+	+ *	+	–	+	+	–

Notes: +, presence of gene; –, absence of gene; *, frame shift or premature stop codon.

## Data Availability

The data are contained within the article and the Supplementary Materials.

## Supplementary Materials

The following are available online at https://www.mdpi.com/article/10.3390/vaccines11020419/s1, Table S1. Proteins encoded by genes of the OAS family in mammalian species investigated in this study. Figure S1. Amino acid sequences of *OAS* family proteins encoded by genes that have been investigated in this study. Figure S2. Phylogenetic tree of species investigated in this study and mapping of gene loss and gain events. Figure S3. Prediction of exons for *OAS1* and *OAS2* genes of the Chinese pangolin (*Manis pentadactyla*). Figure S4. Predicted structure of OAS3 in Pallas’s mastiff bat (*Molossus molossus*). Figure S5. Amino acid sequence alignment of OAS1 and OAS5 proteins.
