# Supplementary material for: Differential Loss of OAS Genes Indicates Diversification of Antiviral Immunity in Mammals
Source: Vaccines (Basel). 2023 Feb 12;11(2):419. doi: 10.3390/vaccines11020419 (PMC9964502; doi:10.3390/vaccines11020419)
Supplement: Supplementary file 1 [file vaccines-11-00419-s001.zip › vaccines-2165586-supplementary.pdf]

Table S1. Proteins encoded by genes of the OAS family in mammalian species investigated in this study

**Species** common name	**Species** scientific name	**OAS1** acc. nr.	**OAS2** acc. nr.	**OAS3** acc. nr.	**OAS4** acc. nr.	**OAS5** acc. nr.	**OASL** acc. nr.	**OASL2** acc. nr.
Human	*Homo sapiens*	NP_058132.2	NP_002526.2	NP_006178.2	n.a.	n.a.	NP_003724.1	n.a.
Bactrian camel	*Camelus bactrianus*	XP_010967910.1	n.a.	n.a.	XP_045368463.1	n.a.	XP_010967907.1	n.a.
Arabian camel	*Camelus dromedarius*	XP_031299144.1	n.a.	n.a.	XP_031304545.1	n.a.	XP_010993548.1	n.a.
Alpaca	*Vicugna pacos*	XP_031526050.1	n.a.	n.a.	XP_031529082.1	n.a.	XP_015095194.1	n.a.
Dog	*Canis familiaris*	XP_038292031.1	NP_001041599.1	NP_001041556.1	XP_038516678.1	n.a.	NP_001240716.1	NP_001041558.1
Malayan pangolin	*Manis javanica*	XP_036862000.1*	XP_017496839.2	n.a.	n.a.	n.a.	n.a.	n.a.
Chinese pangolin	*Manis pentadactyla*	See Figure S1 (Mp_OAS1)	See Figure S1 (Mp_OAS2)	n.a.	n.a.	n.a.	n.a.	n.a.
Chinese rufous horseshoe bat	*Rhinolophus sinicus*	XP_019589691.1	XP_019589650.1	XP_019589648.1	n.a.	n.a.	XP_019583429.1	XP_019583430.1
Pallas's mastiff bat	*Molossus molossus*	XP_036133189.1*	XP_036133188.1	XP_036132196.1	n.a.	XP_036126503.1	XP_036132778.1	n.a.
Greater mouse-eared bat	*Myotis myotis*	XP_036210168.1	KAF6282567.1	KAF6282568.1	n.a.	XP_036199939.1	XP_036209649.1	n.a.
Jamaican fruit bat	*Artibeus jamaicensis*	XP_036989359.1	XP_036989379.1*	XP_036989355.1	n.a.	XP_037010350.1	XP_037012124.1	n.a.

Notes: *, proteins predicted from genes containing reading frame shifts or premature stop codons; n.a., not applicable (no orthologous gene and protein in this species); acc. nr., GenBank accession number; OAS, 2'-5'-oligoadenylate synthetase; OASL, OAS-like.

>NP_058132.2 2'-5'-oligoadenylate synthase 1 isoform 1 [*Homo sapiens*]
MMDLRNTPAKSLDKFIEDYLLPDTCFRMQINHAIDIICGFLKERCFRGSSYPVCVSKVVKGGSSGKGTTL
RGRSDADLVVFLSPLTTFQDQLNRRGEFIQEIRRQLEACQRERAFSVKFEVQAPRWGNPRALSFVLSSLQ
LGEGVEFDVLPAFDALGQLTGGYKPNPQIYVKLIEECTDLQKEGEFSTCFTELQRDFLKQRPTKLKSLIR
LVKHWYQNCKKKLGKLPPQYALELLTVYAWERGSMKTHFNTAQGFRTVLELVINYQQLCIYWTKYYDFKN
PIIEKYLRRQLTKPRPVILDPADPTGNLGGGDPKGWRQLAQEAEAWLNYPCFKNWDGSPVSSWILLAESN
SADDETDDPRRYQKYGYIGTHEYPHFSHRPSTLQAASTPQAEEDWTCTIL

>NP_002526.2 2'-5'-oligoadenylate synthase 2 isoform 2 [*Homo sapiens*]
MGNGESQLSSVPAQKLGWFIQEYLKPYEECQTLIDEMVNTICDVLQEPEQFPLVQGVAIGGSYGRKTVLR
GNSDGTLVLFFSDLKQFQDQKRSQRDILDKTGDKLKFCLFTKWLKNNFEIQKSLDGFTIQVFTKNQRISF
EVLAAFNALSLNDNPSPWIYRELKRSLDKTNASPGEFAVCFTELQQKFFDNRPGKLKDLILLIKHWHQQC
QKKIKDLPSLSPYALELLTVYAWEQGCRKDNFDIAEGVRTVLELIKCQEKLCIYWMVNYNFEDETIRNIL
LHQLQSARPVILDPVDPTNNVSGDKICWQWLKKEAQTWLTSPNLDNELPAPSWNVLPAPLFTTPGHLLDK
FIKEFLQPNKCFLEQIDSAVNIIRTFLKENCFRQSTAKIQIVRGGSTAKGTALKTGSDADLVVFHNSLKS
YTSQKNERHKIVKEIHEQLKAFWREKEEELEVSFEPPKWKAPRVLSFSLKSKVLNESVSFDVLPAFNALG
QLSSGSTPSPEVYAGLIDLYKSSDLPGGEFSTCFTVLQRNFIRSRPTKLKDLIRLVKHWYKECERKLKPK
GSLPPKYALELLTIYAWEQGSGVPDFDTAEGFRTVLELVTQYQQLCIFWKVNYNFEDETVRKFLLSQLQK
TRPVILDPAEPTGDVGGGDRWCWHLLAKEAKEWLSSPCFKDGTGNPIPPWKVPVKVI

>NP_006178.2 2'-5'-oligoadenylate synthase 3 isoform 1 [*Homo sapiens*]
MDLYSTPAAALDRFVARRLQPRKEFVEKARRALGALAAALRERGGRLGAAAPRVLKTVKGGSSGRGTALK
GGCDSELVIFLDCFKSYVDQRARRAEILSEMRASLESWWQNPVPGLRLTFPEQSVPGALQFRLTSVDLED
WMDVSLVPAFNVLGQAGSGVKPKPQVYSTLLNSGCQGGEHAACFTELRRNFVNIRPAKLKNLILLVKHWY
HQVCLQGLWKETLPPVYALELLTIFAWEQGCKKDAFSLAEGLRTVLGLIQQHQHLCVFWTVNYGFEDPAV
GQFLQRQLKRPRPVILDPADPTWDLGNGAAWHWDLLAQEAASCYDHPCFLRGMGDPVQSWKGPGLPRAGC
SGLGHPIQLDPNQKTPENSKSLNAVYPRAGSKPPSCPAPGPTGAASIVPSVPGMALDLSQIPTKELDRFI
QDHLKPSPQFQEQVKKAIDIILRCLHENCVHKASRVSKGGSFGRGTDLRDGCDVELIIFLNCFTDYKDQG
PRRAEILDEMRAQLESWWQDQVPSLSLQFPEQNVPEALQFQLVSTALKSWTDVSLLPAFDAVGQLSSGTK
PNPQVYSRLLTSGCQEGEHKACFAELRRNFMNIRPVKLKNLILLVKHWYRQVAAQNKGKGPAPASLPPAY
ALELLTIFAWEQGCRQDCFNMAQGFRTVLGLVQQHQQLCVYWTVNYSTEDPAMRMHLLGQLRKPRPLVLD
PADPTWNVGHGSWELLAQEAAALGMQACFLSRDGTSVQPWDVMPALLYQTPAGDLDKFISEFLQPNRQFL
AQVNKAVDTICSFLKENCFRNSPIKVIKVVKGGSSAKGTALRGRSDADLVVFLSCFSQFTEQGNKRAEII
SEIRAQLEACQQERQFEVKFEVSKWENPRVLSFSLTSQTMLDQSVDFDVLPAFDALGQLVSGSRPSSQVY
VDLIHSYSNAGEYSTCFTELQRDFIISRPTKLKSLIRLVKHWYQQCTKISKGRGSLPPQHGLELLTVYAW
EQGGKDSQFNMAEGFRTVLELVTQYRQLCIYWTINYNAKDKTVGDFLKQQLQKPRPIILDPADPTGNLGH
NARWDLLAKEAAACTSALCCMGRNGIPIQPWPVKAAV

>NP_003724.1 2'-5'-oligoadenylate synthase-like protein isoform a [*Homo sapiens*]
MALMQELYSTPASRLDSFVAQWLQPHREWKEEVLDAVRTVEEFLRQEHFQGKRGLDQDVRVLKVVKVGSF
GNGTVLRSTREVELVAFLSCFHSFQEAAKHHKDVLRLIWKTMWQSQDLLDLGLEDLRMEQRVPDALVFTI
QTRGTAEPITVTIVPAYRALGPSLPNSQPPPEVYVSLIKACGGPGNFCPSFSELQRNFVKHRPTKLKSLL
RLVKHWYQQYVKARSPRANLPPLYALELLTIYAWEMGTEEDENFMLDEGFTTVMDLLLEYEVICIYWTKY
YTLHNAIIEDCVRKQLKKERPIILDPADPTLNVAEGYRWDIVAQRASQCLKQDCCYDNRENPISSWNVKR
ARDIHLTVEQRGYPDFNLIVNPYEPIRKVKEKIRRTRGYSGLQRLSFQVPGSERQLLSSRCSLAKYGIFS
HTHIYLLETIPSEIQVFVKNPDGGSYAYAINPNSFILGLKQQIEDQQGLPKKQQQLEFQGQVLQDWLGLG
IYGIQDSDTLILSKKKGEALFPAS

>XP_010967910.1 2'-5'-oligoadenylate synthase 1 isoform X1 [*Camelus bactrianus*]
MALRDTRANQLDKFIEDNLLPDTPFRTQVREAVDIICTFLKERCFRGAAHPVRVSKVVKGGSSGKGTTLR
GRSDADLVVFLTNLKSFQEQLRRRGEFIQEITRQLEACQREKRFKVKFEIHNSPRANPRALSFMLTSPQL
DQGVEFDVLPAFDALGQLTRGYRPDPKVYIQLIEECESLGKEGEFSPCFTELQRAFLRDRPTKLKSLIRL
VKHWYQECKQQLGKPLPPQYALELLTVYAWEQGSRKTEFNTAQGFRTVLELVRKHQDLCIYWENYYSLEN
PIIGCYLKKQLGKLRPVILDPADPTGNIGGGDPRSWQRLAEEATAWLSYPCLKKWDGSPVDSWHVPPRKD
SYWTHEACDDCQHWEFSPRPITLQGGRPPLEEENWSCVIL

>XP_045368463.1 2'-5'-oligoadenylate synthase 1-like isoform X1 [*Camelus bactrianus*] = OAS4
MTFSPWIYSRMGVPGVHNCRGSFLIPRCQLRQCGPGASSCHNSSQIRSSLMVAGSTCLLADLKLHLYRKL
HLCNDSDEAQLCALALLPYQVDFVKASVSRVKELIRLMMHWFKTSFASTTEENKFRRLPSSYTVELLTIY
IWERAEKPLFFSLVQGMRAVLKLLVRYAEIDVVWHRHYHRKFPIFVKVYQKHTRPFILDPVNPTINVCDT
CNAWDEVAHVARRSLLKPLFSRVRAEPPWLFTNDW

>XP_010967907.1 2'-5'-oligoadenylate synthase-like protein isoform X1 [*Camelus bactrianus*]
MALPQELFDTPAYKLTTFVQQCLHPSREWKEEVLEVVRTVEYSLRKKCFQRKSRLDKEVWVQKVIKVGSL
GNGTMLGNTTEVELVVFLSCFRSFQEEAKHHQRILNLIYEKLLYCQDLLALQLQDLRLVQGAPCEVVSFT
VQTRETGEPITVTLVPAFGALELYLHKPQPPPEVYVSLIKACNVPGNFSPSFSELQKNFIKHRPAKLKSL
LRLVKHWYLEYVKALCPRANLPPLYALELLTIYAWEVGTQENENFDLDRGLVTVMSLLTKYQSLCIFWTK
YYTFQNTIIEDFVRKELKNERPIILDPADPTHNVARGYKWETVSQRARQCLKQNCCYNNKHQVCSWNVKS
ARDIQVTVEQCGYPDLTLTVNPYKLIKEIKEEIQETLGSSAVLRLSFQEPSGERQLLRSRDYLASYGIFS
NTRICLLETVPPEIQLFVKNPSGWSHSYAVDPNSLILDLKQQIEEKEELFREEQQLEFQGQVLQDWWRLG
ICGFQDSDTLILSKKKAGEAQFLPR

>XP_031299144.1 2'-5'-oligoadenylate synthase 1 [*Camelus dromedarius*]
MALRDTRANQLDKFIEDNLLPDTPFRTQVREAVDIICTFLKERCFRGAAHPVRVSKVVKGGSSGKGTTLR
GRSDADLVVFLTNLKSFQEQLLHRGEFIQEITRQLEACQREKRFKVKFEIHNSSRANPRALSFMLTSPQL
DQGVEFDVLPAFDALGQLTRGYRPDPKVYIQLIEECESLGKEGEFSPCFTELQRAFLRDRPTKLKSLIRL
VKHWYQECKRRNGKEGALPPQYALELLTVYAWERGSGSPEFNTAQGFRTVLELVRKHQDLCIYWENYYSL
ENPIIGRYLKKQLGKPRPVILDPADPTGNVAGRDPGSWWLLAQEAATWLRYPCLTECNGSPVSTWEVQEA

LPARSACTGSPARSIVMALRDTRANQLDKFIEDNLLPDTPFRTQVREAVDIICTFLKERCFRGAAHPVRV
SKVVKGGSSGKGTTLRGRSDADLVVFLTNLKSFQEQLCRRGEFIQEITRQLEACHREKKFKVMFKIHNSS
YANPRALSFALTSPQLDQGVEFDVLPAFDALGQLTRGYRPDPKVYIQLIEECESLGKEGEFSPCFTELQR
AFLRDRPTKLKSLIRLVKHWYQECKQQLGKPLPPQYALELLTVYAWEQGSRKTEFNTAQGFRTVLELVRK
HQDLCIYWENYYSLENPIIGRYLKKQLRKPRPVILDPADPTGNIGGGDPRSWQRLAEEATAWLSYPCLKK
WDGSPVDSWHVPPRKDSYWTHEACDDCQHWEFSPRPITLQGGRPPLEEENWSCVIL

>XP_031304545.1 2'-5'-oligoadenylate synthase 1-like isoform X1 [*Camelus dromedarius*] = OAS4
MTFSPWIYSRMGVPGVHNCRGSFLIPRCQLRQCGPGASSCHNSSQIRSSLMVAGSTCLLADLKLHLYRKL
HLCNDSDEAQLCALALLPYQVDFVKVSVSRVKELIRLMMHWFKTSFASTTEENKFRRLPSSYTVELLTIY
IWERAEKPLFFSLVQGMRAVLKLLVRYAEIDVVWHRHYHRKFPIFVKVYQKHTRPFILDPVNPTINVCDT
CNAWDEVAHVARRSLLKPLFSRVRAEPPWLFTNDW

>XP_010993548.1 2'-5'-oligoadenylate synthase-like protein isoform X2 [*Camelus dromedarius*]
MALPQELFDTPAYRLTTFVQQCLHPSREWKEEVLEVVRTVEYSLRKKCFQRKSRLDKEVWVQKVIKDLLA
LQLQDLRLVQGAPCEVVSFTVQTRETGEPITVTLVPAFGALELYLHKPQPPPEVYVSLIKACNVPGNFSP
SFSELQKNFIKHRPAKLKSLLRLVKHWYLEYVKALCPRANLPPLYALELLTIYAWEVGTQENENFDLDRG
LVTVMSLLTKYQSLCIFWTKYYTFQNTIIEDFVRKELKNERPIILDPADPTHNVARGYKWETVSQRARQC
LKQNCCYNNKHQVCSWNVKSARDIQVTVEQCGYPDLTLTVNPYKLIKEIKEEIQETLGSSAVLRLSFQEP
SGERQLLRSRDYLASYGIFSNTRICLLETVPPEIQLFVKNPSGWSHSYAVDPNSLILDLKQQIEEKEELF
REEQQLEFQGQVLQDWWRLGICGFQDSDTLILSKKKAGEAQFLPR

>XP_031526050.1 uncharacterized protein LOC102530627 [*Vicugna pacos*] = OAS1-like
MDSFPTGQLTRDYRPDPKVYIQLIKECESLGKEGEFSPCFTELQRAFLRDRPTKLKSLIRLVKHWYQECK
SRNGKKRALPPQYALELLTVYAWERGSRSPEFNTAQGFRTVLELVLKHQDLCIYWEKYYSFETPIIGDYL
KKQLGKPRPVILDPADPTGNVAGRDPSSWQLLAQEAAVCLGYPCFREWNGSPVSAWKVQCWYETQAPESS
TPDTPGTRPHSGQLPTEERELKRTKVEATEMRVEASEMCCLRCLGPRLHQGAQCDVLPASHALGQLTRGY
RPDPKVYIQLIKECESLGKEGEFSPCFTELQRAFLRDRPTKLKSLIRLVKHWYQECKRQRGKPLPPQYAL
ELLTVYAWEQGSGKTEFNMAQGFRTVLELVLKHQDLCIYWEKYYSFETPIIGDYLKKQLGKPRPVILDPA
DPTGNIGGGDRHSWQRLAEEAAAWLSYPCLKKWDGSPVDSWHVLPRKDSYWTHEACDDGQHWEFSPRPIT
LQGGRPASEEENWTCVIL

>XP_031529082.1 2'-5'-oligoadenylate synthase 1-like isoform X1 [*Vicugna pacos*] = OAS4
MGVPGVHNCRGSFLIPRCQLRQCGPGASSCHNSSQIRSSLMVAGSTCLLADLKLHLYRKLHLCNDSDEAQ
LCALALLPYQVDFVKASVSRVKELIRLMMHWFKTSFASTTEENKFRRLPSSYTVELLTIYIWERAEKPLF
FSLVQGMRAVLKLLVRYAEIDVVWHRHYHRKFPIFVKVYQKHTRLFILDPVNPTINVCDTCNAWDEVAHV
ARRSLLKPLFSRVRAEPPWLFTNDW

>XP_015095194.1 2'-5'-oligoadenylate synthase-like protein isoform X1 [*Vicugna pacos*]
MALPQELFDTPASKLTAFVHHCLHPSQEWKKEVLEVVRTVGHSLRKNCFQRKSSLDKEVRVQKVIKVGSF
GNGTVLRNTTEVELVVFLSCFRSFQEEAKHHQRILNLIYEKLLYCQDLLALQLQDLRLVRGNPCEVVSFT
VQTRETGEPITVTLVPAFGALKPCHPKYQPPPEVYVSLIKACNVPGNFSPSFSELQKIFIKHRPAKLKSL
LRLVKHWYLEYVKALCPRANLPPLYALELLTIYAWEVGTQENENFRLDRGLVTVMSLLKEYQSLCIFWTK
YYTFQNTIIEDFVRKELKNERPIILDPADPTHNVARGYKWETVAQKARQCLKQNCCCDNKDRVCSWNVKS
TRDIQVTVEQWGYPDLTLTVNPYEPIKKVKEEIQETLGSSAVLRLSFQEPSGERQLLRSRDSLASYGIFS
NTRICLLETVPPEIQLFVENPSGWSHSYAVDPNSLILDLKQQIEQKEGLFIEEQQLEFQGQVLQDWWRLG
ICGFQDSSTLILSKKKAGEAHFLPR

>XP_038292031.1 2'-5'-oligoadenylate synthase 1 isoform X1 [*Canis lupus familiaris*]
MPELKDTPAKDLDRFIENYLLPDTQFRRQVKEAIHIISTFLKERCFQGAAHPVRVSKVVKGGSSGKGTTL
RGRSDADLVVFLNNLKSFQEQLEKRGQFIWEIKRQLEACQREETFEVYFEVQSLQWEKPRALSFVLKSPQ
LGEGVEFDVLPAFDVLGQWTNNHRPNPEVYIKLIQECENRGTEGEFSTCFTELQRDFLRQRPTKVKSLIR
LVKHWYQKCKQKLGKPLPPQYALELLTVYAWERGNHQTEFITAQGFQTVLKLVLNYQQLCIHWTKYYNFE
TPIIKQYLMRQLAKPRPVILDPADPTGNVAGRDTYGWQRLAQEARVWLSYPCFKKRDGSPVGSWDVLLEE
DYEDNWITCEHRTYSYHDYGWRPVSSGSPNTGMTQSIPQQEENWMCTIL

>NP_001041599.1 2'-5'-oligoadenylate synthase 2 [*Canis lupus familiaris*]
MGIWGSHLYSVQPERLDELIQNSLRPYGECQKQIDDAVDTICAALQETEQIPTVLSVVQGGSYGRKTVLR
GNSDGMIVIFVSDLEQFQDQKRSQDKILNKIWQLLKACQLAMKLPAKMEVQRFRDGLIFQLSTKWQSITF
KVLPAYNALGLSEKPSSQIYRELKRALDMTKAHPGEFSICFTELQQKFFHKYPRKLKDLILLVKYWYQQC
QEKLKGSPLLTMYALELLTVYAWEQGCGAENFDIAEGIRTVLGLIRQQEQLCVYWIVNYNFENETVRNIL
LSQLRSSRPVILDPTDPTNNVSKDNASWQLLKQEAQSWLSALSPNESPGPSWNVLPAPLYATPGHLLDKF
IKDFLQPNKNFLGQIAVAVDFICKFLQKNCFQHSATKVQKTVKGGSTGKGTALKTGSDADLVVFPDSLKS
YTSQKNERCRIIKEIHKQLVACQQEKEFAVKFEISKWKAPRVLSFSLKSKVLNESVNFDVLPAFNVLGQV
NSGSPPSPKIYTELINLYKSSNAEGGEFSTCFTELQCSFVASRPTKVKDLIRLVKHWYKQCERKLKKKGS
LPPKYALELLTIYAWEQGSGAENFDTAEGFRTVLELVTKYQQLCVFWTVNYNFDDETVRNFLLTQIQKTR
PVILDPAEPTGDVGGGDRWCWHLLAKDATEWLSSFCFKDGTGYPVQSWKVPVRII

>NP_001041556.1 2'-5'-oligoadenylate synthase 3 [*Canis lupus familiaris*]
MDVYRTPAAALASLVARRLQPSAEFQRAAWRALGALATTLRERGDRAAAQPWRVLKTAKGGSAGRGTALR
GGCDSEIVIFLDCFKSYKDHSVDRAEILKDLWDLLQSWWQKPIPGLNFETLWQDRPGVLQFRLASTDLEN
WMDVSLVPAFDALGQLCAGAKPAPQVYSTLLHSGCQGGEHAACFAELRRNFVNVRPAKLKSLILLVKHWY
RQVCQEEAKREMLPPAYALELLTIFAWEQGCGKDAFSLAQGLRTVLGLIQEYRQLCVFWTLNYGFENPTV
RSFLSSQLKKPRPVILDPADPTWDVGNGATWHWDILAREAESCYEHPCFLQTAGDTVQPWEGTGLPRAGC
SGLDHPIQRDDAQRTPGNSSSLNAVPPRAGSRQPSWPAPRPPGPDSITPSTLGRAVDLSQIATKDLDRFI
QDHLKPNPQFQKQVGKAINVILGCLREKCVYKASRVSKGGSFGRGTDLRGGCDAELVIFLNCFEDYRDQR
ARRPEILQEMQAQLESWWQDPVPGLSLEFPEQTVPEALQFRLVSTALESWMDVCLVPAFDAVGQLCAGAK
PAPQVYSTLLQSGCQGGEHAACFAELRRNFVNVRPAKLKSLILLVKHWYRQVAAQNKGQQPACASLPPVY

ALELLTIFAWEQGCGEDSFKMAQGLKTVLELVQQHQQLCVYWTVNYSFEDPAIRTHLLGQLQKPRPLILD
PGDPTWNVGQGSWELLAQEAAVLETQACLRSTEGTSVQPWDVMPALLYQTPAGDLDKFISDFLQPNRQFL
AQVNKAVDTICSFLKENCFQNSAIKVLKVVKGGSLAKGTALRGRSDADLVVFLSCFSQFAEQGNRRAEII
SEIRAQLEACQQKMQLEVKFEIPKRENSRVLSFSLKSQTMLDQSVDFDVLPAFNALGQVVSSYRPPSQVY
VDLIYSYNNAGEYSTCFTELQRDFIISRPTKLKSLIRLVKHWYRQCNKMPRGRGSLPPQHGLELLTVYAW
EQGGQSAQFNMAQGFRTVLELVSQYRQLRVYWTVNYDNEDQTVRDFLSRQLRQPRPIILDPADPTGNLGH
NARWDLLATEATACMSALCCTDRDGTPIQPWPVKAAV

>XP_038516678.1 2'-5'-oligoadenylate synthase 3-like isoform X1 [*Canis lupus familiaris*] = OAS4
MWALKCYKCECCGSDFVSCKALKYHFRQKHFGKAPCEKCGQEVLRENLQDHSKSVYDRSAMERHKLNWEN
LEERNIKHMATEISKTCRMCSRHFGTVFSRKNHEIQEHHFTANKRAQMSIGFSPQNMLECKSPQELQRFA
DENIRPAPGALRAACVAEVGALLQLLRACFPVPASRVIQGGSYVKGTDTQGRSEIDVVLLSDVFANVNHC
KKQLREGLDALRENLKQTSRGNRILMGKRASLSLRFNFLCAEGLHSHSFEIMACCDVLGPAPSTDLRLHL
YRQLYLCHDSEMAQLWALALLPYQVDFVKASVMRVKELIRLMVHWFRTSFANSTEENKFRRLPSSYTVEL
LTIHIWELAGKPLLFSLVQGMRAVLKLLVRYAEIDVVWHRHYHPKFPIFVKVNQKHTRPFILDPANPTVN
VCDTCNAWDEVALVARHSLLKPLFSRVRAEPPWLFTNNW

>NP_001240716.1 2'-5'-oligoadenylate synthase-like protein [*Canis lupus familiaris*]
MAQAMELYDTPASKLDSFVAQWLQPHRSWKEEILEAVKTVQQFLREEHFEGDYGPDQEVRVLKVVKVGSF
GNGTVLRNTLEVELVVFLSCFHSFQQEAEHHQAILSLIQKKLWCCRDLLALGLEDVEIIQGVPDALVFTI
QTRRTAEIITVTIVPAYRALGPSASNSQPYPEVYESLIEAQGFPGNFSPSFSELQRNFVKHRPTKLKSLL
RLVKHWYLQYVKAKCPRAALPPDYALELLTIYAWEMGTQEDESFRLDEGFTTVMELFQEYEFLCIYWTKY
YTFQNPVIEDFVRKQLKRDRPIILDPADPTHNVAEGYRWDIVAQRARQCLKQDCCYDNKEKPVPSWNVKK
ARDIQVTVEQWGYSDLILRVNPYKPIKKIQEKMWQSRCCSGLQHLYLQELGAKQQLLSSQYSLADYGVFS
NTRICLVETNSHEIQVFVKNPDGGSDAYTTDAKGFILGLKQQIEYKQGLPRKQQQLEFQGQVLKDWLPLQ
NYGIQHRDTLILSKKKAERFPFLPR

>NP_001041558.1 54 kDa 2'-5'-oligoadenylate synthase-like protein 2 [*Canis lupus familiaris*]
MALVQKLYETPAERLLAFVERSLQPEGDWKEEVKDAWQRIERFFRDQCFRDELVLDQEVRVLKVVKGGSS
GKGTTLNYSSDVDLVLFLSCFSSFQDQAAHRRSIISFIKKRLIQHSKNLAYSITIVPQKETTRVPRSLSF
QVQARKTSEVIGVDVLPAYDALGHFSSDFKPSPEIYEDLITSGGPPGEFSPSFTELQRHFVKSCPVKLKN
LLRMVKHWYLQCLKPKYRNAALPPKYAFELLTIYAWEIGTDKSDNFNLDEGFRAVMELLLDYEDICRYWT
KYYDFQNETVRIHIKQQLKECRPVILDPADPTNNLGSEKRWDLVAKEAARCLRQACCRTEDPSQGWHVQP
ARNVQVLVKKAGEEPQTFSVDPHSPIWKMKAEIKRTFGFYGQQRLSFQEPGGERQLLSSQQTLAHYGIFS
KVNIRVLETFPSEIQVFVKDSSGQSKPYAIYPEDSIRDLKEKIEEAGGPYVEDQILKFQNRRLWNHRSLS
DLQIKDCDTITLIRRSHRSPGLPMIRLFT

>XP_036862000.1 LOW QUALITY PROTEIN: 2'-5'-oligoadenylate synthase 1-like [*Manis javanica*]
MGQLRDIPATHLDKYIEDQLLPDMHFRVQVNQAIDIISSFLKERCFQSAAHSVRVSKVVKGGSXGKGTSL
RGRSDADLVVFLSPPTSFQEQLQHRGEFVREIRRQLEACQREKKFDVLFEVQNPRWEKPRALSFMLRSPR
IQEGVEFDILPAFDVLGQLTSDYRPDPQIYVRLMQESQRLGRGGEFSTCFTELQRAFLKQRPTKVKSLIR
LVKRWYQECKKELGKPLPPQYALELLTIYAWEKGSMDTEFITAQGFLTVLKLVLNYQQLCIYWTKYYTLE
DPFIKRYVMAQLGKPRPIILNPADPTGNVAGGEPNSWPRLAQKARAWLSYPCFKKWDGSSVGSWDVQPKD
NLCSIP

>XP_017496839.2 2'-5'-oligoadenylate synthase 2 isoform X1 [*Manis javanica*]
MGNWESRLYSVPPQKLGEFVQNSLRPFEDCQNKIDKTVNTICAVLQEAEQFPLVISVAKGGSYGRKTVLR
GNSDGTLVVFASDFEQFQDQTKSPSEILHKIRDVLRVWQLTTELAAQVEVQMVSGTLTIQLSTKRQSITF
KVLPAFNALGLSDKPSTWTYLELKRALDQTHSNPGEFSVCFTELQQKFFKNHPRKLKDLILLVKYWYEQC
QEKLKYAPLPPVYALELLTVYAWEQGCRTEDFDIVEGIRTVLGLILQQEQLCVYWVINYNFENVTIRNIL
LSQLRSSRPVILDPADPTNNVSKDKMCWQLLKEEARIWLSSSGLNESPGLSWNVLPAPLYTTPGHLLDKF
IKDFLQPNKIFLEQIERAVKIIRTFLQHNCFQHSTTKIQKIVQGGSTAKGTALKNGSDADLVVFPDWLNS
YTSQKNERSRVIREIHKQLQACQQKLDLEVKFEISKWKAPRVLSFSLKSKVLNESIDFDVLPAFNVLGHK
NSHTAPSPSVYSEHINLYSSSDALGGEFSTCFTELQRDFVLSRLTKLKDLIRLVKHWYRQCEKKLKQKGN
LPPKYALELLTIYAWEQGSGTTNFNTAEGFRTVLELITKYQQLCIFWTVNYNLEDETMRNFPLTQIQRTR
PVILDPAEPTGDVGGGDRWCWHLLAKEATEWMSSLCFKDGTGEPVQAWKVPTVQTPGSCGAGMYPITQEM
FSFRICRTLE

>Mp_OAS1 [*Manis pentadactyla*] – corrected prediction, partially identical to XP_036785080.1
MGELRDIPATHLDKYIEDQLLPDTHFRVQVNQAIDIISSFLKERCFQRAAHSVRVLKVVKGGSSGKGTSL
RGRSDADLVVFLSPLTSFQEQLQHRGEFVREIRRQLEACQREKKFDVVFEVQNPRWEKPRALSFMLRSPR
IQEGVEFDVLPAFDVLGQLTRDYRPDPQIYIQLIQESQCLGRGGEFSTCFTELQRAFLKQRPTKVKSLIR
LVKRWYQECKKELGKPLPPQYALELLTVYAWEKGSMETEFITAQGFLTVLKLVLNYQQLCIYWTKYYTLE
DPFIKRYMMAQLGKPRPVILDPADPTGNVAGGEPNSWPRLAQEARAWLNYPCFKKWGGSSVGSWDVQ
PRDNLCSIP

>Mp_OAS2 [*Manis pentadactyla*] – corrected prediction, identical to part of XP_036785080.1
MGNWESHLYSVPPQKLGEFVQNSLRPFEDCQKKIDETVNTICAVLQEAEQFPLVISVAKGGSYG
RKTVLRGNSDGTLVVFASDFEQFQDQTKSPSEILHKIREVLRVWQLITELAAQVEVQMVSGTLTIQLSTK
RQSITFEVLPAFNALGFSDKPSTWTYLELKRALDQTHSNPGEFSVCFTELQQKFFKNHPRKLKDLILLVK
YWYEQCQEKLKYSPLPPVYALELLTVYAWEQGCRTEDFNIVEGIRTVLRLILQQEQLCVYWVVNYNFENV
TIRNILLSQLRSSRPVILDPVDPTNNVSKDKMCWQLLKQEAQIWLSSSGLNESPGLSWNVLPAQLYTTPG
HLLDKFIKDFLQPNKIFLEQIERAVKIIRTFLQHNCFQHSTTKIQKIVQGGSTAKGTALKNGSDADLVVF
PNWLNSYTSQKNERSRVIREIHKQLEACQQKLDLEVKFEISKWKAPRVLSFSLKSKVLNESIDFDVLPAF
NVLGHKNSHTAPSPSVYSELIDLYSSSDALGGEFSTCFTELQRNFVISRPTKLKDLIRLMKHWYRQCERK
FKQKGNLPPKYALELLTIYAWEQGSGTTNFNTAEGFRTVLELITKYQQLCIFWTVNYNLEDETMRNFLLT
QIQRTRPVILDPAEPTGDVGGGDRWCWHLLAKEATEWMSSLCFKDGTGEPVQAWKVPTVQTPGSCGAGMY

PVTQEMFSFRICRTLE

>XP_019589691.1 PREDICTED: 2'-5'-oligoadenylate synthase 1 isoform X1 [*Rhinolophus sinicus*]
MELRNTPAKNLDKFIQDHLLPDKHFRSQVKQAIDSICSFLKEKCFQHASHSVRVSKVVKGGSSGKGTTLR
GRSDADLVVFLSPLRSFQEQFDRRGEFIQEIRKQLEACQREETFAVEFEVQNHRWTNPRALGFVLKSPTI
RDGVEFDVLPAFDVLADQVTDNYIPDPQIYVDLIQACEHLKKEGEFSTCFTELQRAFLRQRPTKLKSLIR
LVKHWYQTFKEKCGESLPPQYALELLTVYAWERGGEKPEFNTAQGFKTVLELVRGYQQLWIYWPKYYNFK
NPVICHYLMKQLEKPRPVILDPADPTGNVGGGDRRRWQRLAQEAETWLSYPCFKNHDGCPVDSWDIQF

>XP_019589650.1 PREDICTED: 2'-5'-oligoadenylate synthase 2 isoform X1 [*Rhinolophus sinicus*]
MGNSAPSSMPTQELDAFIQESLRPYEGCQKQIDKAVDTICAALHEAEEFLVTDVAKGGSYGRETVLRDHS
DGTLVIFISDLQTFTDQKKMRYEILHKIWNWLKHCQLERKLAAKILMSSSGLVVELSTRWQSITFEVLPA
FNALGLSEKPSPWTYRELKRSLDMTKASPGEFSVCFTKLQQNFFNNHPRKLKDLILLVKYWYQQCQKKLR
ALPLLSSYALELLTVYAWEQGCGAEDFEIVEGIRTVLGLIKQSRQLCVYWTVNYNFEDETVRNIVLGQIR
SPRPVILDPTDPTNNVSKDSICWQLLKEEAEMWLSCLNESPGPSWNVLPASLQETPGHRLDKFIKDFLQP
SEAFLNRIHKAVDIICSFLKENCFRYSTTKVQKAVKGGSAGKGTTLKTGSDADLVVFTTSLKSYTSQNTE
RCDIIKEIRKQLEVCQQQERFEVKFEISKWKAPRVLSFSLKSKEFDESVDFDVLPAFNALDKWKPGSTPS
PKVYRDLICLYKSSNIVGGEFSTCFTELQRDFIISRPTKLKDLIRLVKHWYKQCEKKLKKKGSLPPKYAL
ELLTIYAWEQGSGLADFDTAEGFRTVLELIRRYQQLCIFWTVNYNFEDATVRDFLLTQLQKTRPVILDPA
EPTGDVGGGDRWCWYLLAKEATEWLSFPCFIDGAGHPVQFWKVPTAQTPGSCGARLHPVVDEMFSFRSRQ
ILQ

>XP_019589648.1 PREDICTED: 2'-5'-oligoadenylate synthase 3 isoform X1 [*Rhinolophus sinicus*]
MDLYRTSATELDRLVISLQPPTEFVETARRALGALGAALRERRGLRDAAAPTWRVLKIAKGGSSGRGTAL
RGGCDSELVIFLDCFKSYKDQRLHRPKILKEMQALLKSWCQHPVPGLSLQFPQQDTPEVLQFRLASTDLE
NWMDISLVPAFDALGQLRSNVKPKPQVYSALLDSGCHAGEHAACFAELRRNFVNTRSAKLKNLILLVKHW
YRQVCPEETRREMPPDYALELLTIYAWEQGCKKDAFSLAQGLRTVLGLIQQYQSLCVFWTINYDFEDPAV
KRFLQRQLERPRPVILDPADPTWDVGNGAACNWNLLAQEAESCYDRPCFLQAAGGAVEPWKVPGLPQSGL
DDHVLQDPVQKSLKDSCSLDAVQPRPGSRQPSCPAPAPLEAASIPPCPPGMVSDLSQISAKELDRFIQDH
LKPNPQFQKLVSKAIDVILGRLLQKCVHKASRVSKGGSFGRGTDLRGGCDAELVIFLNCFKTYTDQGTYR
SEILDDMRAQLESWWQDPVPGLSLKFPNQTVPEALQFQLVSTALESWMHVSLLPVFDAVGQLSSGAKPEP
QVYSTLLDSGCQEGEHAACFAELRRNFVNTRPVKLKNLILLVKHWYHQVAAQNKEERPSLPPAYALELLT
IFAWERGCGEDRFSMAQGLRTVLGLVRKHQQLCVFWTVNYSFEDPALRTHLLGQLRKPRPLVLDPADPTW
NVGQGCWKLLAQEAAALEMQACLMGTKGTPVQPWDVMPALLYQTPAWDLDKFISEFLQPDRQFLTQVNKA
VDTICAFLREKCFQNSPIKVLKVVKGGSSAKGTALRGRSDADLVVFLSCFSQFTEQGNRRADIISEIRAQ
LEACQQEKQFEVKFEISKWENPRVLSFSLASQTMLDQSVDFDVLPAFDALGQLVSRSRPSPQVYVDLIHS
FHNAGEYSSCFTELQRDFIVSRPTKLKSLIRLVKHWYQQCNKMPKGKGSLPPQHGLELLTVYAWEQGGKD
PQFNMAEGFRTVLELVTQYRQLCVYWTINYNRENKTIKDFLEQQLRKPRPIILDPADPTGNLGHNARWDL
LAKEATACMTALCCMGRDGNPIQPWPVKAAV

>XP_019583429.1 PREDICTED: 2'-5'-oligoadenylate synthase-like protein [*Rhinolophus sinicus*]
MALTPDLYGTSASRLNSFVAQYLQPSREWKEEVLEAVRTVEQFLREETFQGEHGLNQDVRVLKVVKVGSF
GNGTVIRSNTEVELVVFLSCFHSFQEEAKHHQTALRLIRKRLWLCQDLLALGLEVLGLVQGIPDVLVFTI
HTKDTAEPITVTIVPAYRALGSSVTSSQPHPEVYENLIEACGYPGNFSPSFCELQRNFVKHRPTKLKSLL
RLVKHWYQQYVKAKCRKAMLPPLYTLELLTIYAWEMGTHGDENFNLDEGLTTVMELLKEYEFLCIYWTKN
YTFQNPIIENFVRKEFKRQRPIILDPADPTHNVAEGYRWDIVAQRASQCLKQDCCYDQENPVPSWNVKSA
RDIWVTVEQWGYPSLILVVNPYEPIRKVKEKIRLSRGSLGLQRLSFQTPGSERQLLSSHNCLADFGIFLD
TPICLLETVFPEIQVFVKNPDGGSHAYAIDPNSFIVALKQQIEDKQGLLIKQQQLEFQGQQLQDWLAFTS
YGIEDSDTLILSEKKAREVPFPYS

>XP_019583430.1 PREDICTED: 2'-5'-oligoadenylate synthase-like protein 2 [*Rhinolophus sinicus*]
MEPSRSLYETPADELDTFVRQSLQPQRAWKEEVQDAWDRSERFLRDVCFHDELIGDQTIRVLKVVKGGSS
GKGTTLNHSSDVDLILFLSCFSSFQHQAWLCESILNIIEEKLDHCSRSLAYNITVAQHRAGSRASRSLSF
LVQARKSSDVIKVDVLPAFDALGNISPDCKPRPEIYENLITVGGRPGQFSPSFTELQRRFVKSRPIKLKN
LLRLVKHWYLQFLKRKYKTPGLPPKYALELLTIYAWEMGTDESENFNLNEGFIAVMELLQDYEKICIYWT
KYYDFQNEVVRNFIKQQLKEPRPVILDPADPTNNLGREKRWDLVAKEAAYCLRQACCSTEDSCQDWDVQP
ARNVQVVVKQTGNEPWKLSVDPYSPIWKMKPEIKRTFGLTGQQRLSFQEPGGERQLLSSKRTLADYGIFS
KVTVQVLETFHDEIQVFVKNCSGHSKPYAICPDDSVRDLKEKIEDAGGPYVEDQILKFQGRRLRNHCSLS
ELQIEDSDTIMLVRRGHSSS

>XP_036133189.1 LOW QUALITY PROTEIN: 2'-5'-oligoadenylate synthase 1 [*Molossus molossus*]
MANSFLAAICPRPSPSLPSPVPSAHRLPADAGCIARARRCRGGGLDKFIKVHLLSDECFRTQVKEAIAII
CSFLKERCFRQSALSPVHVSKVVKGSPPGGSSGKGTTVRGRSGADLVVFLSXLQSFRSSLIREDFIREIK
KQLRACQKEKRFTVYSEIPNNXDPRALSFKLKSSCLQKEVGFDVLPAYDVRGPVSVMGDYRPDPQVCVKL
IQERKSLGQEGEFPSCFWELRRAFLEQRPXLQSPLRLVKRQKCKGKLGQPLPPQYXERTDFITAQGFQSV
WELVLNYQKLCIYXTKYHNTGHPVIXPCLQRQLGKHRPVILDPADPAGNLADGELGGWQRLAQEARVWLS
YPCSRNQDGSPVGSWKIQVYGSSHSRPRGHFDVAYKQHCCSLTMWIIHLCVF

>XP_036133188.1 2'-5'-oligoadenylate synthase 2 [*Molossus molossus*]
MENWLFGAYPKTVYSVPAQKLGEYVQTSLRPSEHCQKQIDATVDTICAALQEVMAIDVAKVPRGRSLNLP
ELQSLSLSKILLPTGDPCDPPKVSRVSHRLLCRWQGGSYGRKTVLRGNSDGTLVIFTSDLGQFRDQKKSQ
HEILNTIWEQLKACQCERKPTAKMEVQRPHGGLAIRLSTWSQSVTFDVLPAFDALGSRDRPSPWTYRELK
RSLDMTKASPGEFSVCFTKLQQKFFNNHPRKLQDLILLVKDWYQQCQKQSASPSWPPSYALELLTVYAWE
QGCGAEDFDIVAGLRTVLGLIKQQEQLCVYWTVNYNFEDETVRNIMLHQIRSSRPVILDPTDPTNNVSKD
QLCWQLLKEEAQRWLSDPSLQEHPGPPWNVLPVPLHETPGHLLDKFIKDFLQPNQTFLSQISTAVDIICA
FLRENCFRRSTTKLQKPVKGGSFSKGTTLKTGSDADLVVFVDSLESYASQRDERCKVIKEIQAQLEAFQR
QAASEQELEVKFEISKWKAPRVLSFSLKSKQLNERVDFDMLPAFNALDKLKPRWRVYTELISLYKNTDTP
GGEFSPCFTELQRDFVMSRPTKLKDLIRLMKHWYKQCERRLKKKGSLPPKYALELLTIYAWEQGSGMPKF

DTAEAFRTVLGLVTRYQHLCIFWTVNYDFENKIVRDFLLTQIQKPRPVILDPADPTGDVGGGNRWCWHLL
ANEAAEWLCSSLCCKDRAGDPVQSWTVPTVQMPGSCGVCTAPVVNEMLSYRSRGVLD

>XP_036132196.1 2'-5'-oligoadenylate synthase 3 isoform X1 [*Molossus molossus*]
MDVYRTPAASLDALVVGSLQLPAEFVGTTRRALGTLETALRECGSRPGPGAAAPPWRVLKTAKGGSFGRG
TALRGGCDSEIVVFLNCFKSYEDQRARRAETLTEMRALLESWRQSPVPGLSLEFLQQDTPGVLQFRLASA
DLENWMDVSLAPAFDALGQLSSGTKPDPQIYSTLLDSGCQDGEHAACFAELRRNFTNTRPTKLKNLILLV
KHWYRQVCPQERRREMPPDYALELLTIFAWEKGCGKDAFSLAQGLRTVLGLIQQHQHLRVFWTTNYGFED
PAVKKFLLRQLDRPRPVILDPADPSWDVGNGAAWHWDLLAREAESCYDSPCFLQAAGGAVQPWEVPGLPL
TAPPILRDPAQKTPEDSSSLDAVHPRAGNGQPSCPDPDPSVVAAIAPCALEVVSDLSQIPARELDRFIQD
HLKPQPQFQKQVSKAFDVILSCLRENGAHKASRVSKGGSFGRGTDLRGGCDAELIVFLNCFKNYKDQGPL
CAEVLRDLRAQLESCGQEPVPGLTLKFAAQTTTKALRFQLVSSALKSWMDVSLLPVFDAVGQLSSGTKPD
PQIYSTLLDSGCQDGEHAACFAELRRNFVNSRPVKLKNLILLVKHWYRQVCPQERRREMPPDYALELLTI
FAWEKGCGKDAFSLAQGLRTVLGLIQQHQHLRVFWTTNYGFEDPAVKKFLLRQLDRPRPVILDPADPTWD
VGNGAAWHWDLLAREAESCYDSPCFLQAAGGAVQPWEVPGLPLAAPPILRDPAQKTPEDSSSLDAVHPRA
GNGQPSCPDPDPSVVAAIAPCALEVVSDLSQIPARELDRFIQDHLKPQPQFQKQVSKAFDVILSCLRENG
AHKASRVSKGGSFGRGTDLRGGCDAELIVFLNCFKNYKDQGPLCAEVLRDLRAQLESCGQEPVPGLTLKF
AAQTTTKALRFQLVSSALKSWMDVSLLPVFDAVGQLSSGTKPDPQIYSTLLDSGCQDGEHAACFAELRRN
FVNSRPVKLKNLILLVKHWYRQVCPQERRREMPPDYALELLTIFAWEKGCGKDAFSLAQGLRTVLSLIQQ
HQHLRVFWTTNYGFEDPAVKKFLLRQLDRPRPVILDPADPTWDVGNGAAWHWDLLAREAESCYDSPCFLQ
AAGGAVQPWEVPGLPLTAPPILRDPAQKTPEDSSSLDAVHPRAGNGQPSCPDPDPSVVAAIAPCALEVVS
DLSQIPARELDRFIQDHLKPQPQFQKQVSKAFDVILSCLRENGAHKASRVSKGGSFGRGTDLRGGCDAEL
IVFLNCFKNYKDQGPLCAEVLRDLRAQLESCGQEPVPGLTLKFAAQTTTKALRFQLVSSALKSWVDVSLL
PVFDAVGQLSSGTKPDPQIYSTLLDSGCQDGEHAACFAELRRNFVNSRPVKLKNLILLVKHWYRQACPQE
RRREMPPDYALELLTIFAWEKGCGKDAFSLAQGLRTVLSLIQQHQHLRVFWTTNYGFEDPAVKKFLLRQL
DRPRPVILDPADPTWDVGNGAAWHWDLLAREAESCYDSPCFLQAAGGAVQPWEVPGLPLTAPPILRDPAQ
KTPEDSSSLDAVHPRAGNGQPSCPDPDPSVVAAIAPCALEVVSDLSQIPARELDRFIQDHLKPQPQFQKQ
VSKAFDVILSCLRENGAHKASRASKGGSFGRGTDLRGGCDAELIVFLNCFKNYKDQGPLCAEVLRDLRAQ
LESCGQEPVPGLTLKFAAQTTTKALRFQLVSSALKSWVDVSLLPVFDAVGQLSSGTKPDPQIYSTLLDSG
CQDGEHAACFAELRRNFVNTRPVKLKNLILLVKHWYRQVAAQNEGGRPACASLPPAFALELLTIFAWEQG
CGKDRFSMAEGLRTVLRLVQQHQQLSVFWTVNYSFEDPALRTHLLGQLRKPRPLILDPADPTWNVGQGSW
ELLAREAAALETQACITSSEGTPVPPWDVMPTLLRQTPASDLDKFISALLQPNQQFLDQVAKAVDTICSF
LRENCFRNSPTKVLKVVKGGSSAKGTALQGRSDADLVVFLTCFSQFTEQGSNRAEIISEIRAQLEACKQE
LQFDVKFEISKWENPRVLSFSLTSPTMLDQSVDFDVLPAFDALGQLGSGSKPPAHVYVDLIRSYNNAGEY
ACCFTELQRDFIVSRPTKLKSLIRLVKHWYQQCNKMPKGKGSLPPQHGLELLTVYAWEQGGQDPQFNMAQ
GFRTVLELVTQYRQLCVYWTVNYSWEDESIKDFLKLQLQKPRPIILDPADPTGNLGHNARWDLLAREAAA
YISALCCMDKDGTPVQPWPVKAAV

>XP_036126503.1 2'-5'-oligoadenylate synthase 1-like [*Molossus molossus*] = OAS5
MQLRDTQARFLDKFIEENLLPDTPFRLQVRQAIHHICGFLKEECFQGAPHRVRVSKVVKGGSSGKGTTLR
GRSDADLVVFLSPLTSFQEQFDRRGEFIQEIKKQLQAFQRETGENFEIRDSGWQNPRVLSFVFSSPMLND
SVEFDVLPAFDVLGQLTGPPDPKIYVQLIDECTRLGKEGEFSPCFTELQRDFLKQRPTKLKSLIRLVKHW
YQVCKEKLGKPLPPQYALELLTVYAWERGSNRSEFSTAQGFRTVLELVMNYRMLCIYWSKYYNFENPVIG
AYLRKQLQKPRPVILDPADPTGNVAGGHPQSWPRLAQEALAWLSYPCFKNWDGSPVGSWNI

>XP_036132778.1 2'-5'-oligoadenylate synthase-like protein [*Molossus molossus*]
MALFPELYGTPASRLDSFVAQWLQPSREWKEEVLEAVRTVEQFLREETFQGERGLDQEIRVLKTVKVGSF
GNGTVLRSNTEVELVVFLSCFRSFQEEAKHHQAVLRLIQKKLWTSPDLLALGLEVLGVFQGVPDALVFTI
QTRETAEPITVTIVPAFRVLGPSVPNSQLHPGVYVSLIGANGFAGNFSPSFCELQRNFVKHRPTKLKNLL
RLVKHWYLQYVKARCPKAMLPPLYALELLTIYAWEMGTQENESFRLDEGLTTVMELLQEYESLCIYWTRH
YTFENPVIEDAVRKQLQRERPIILDPADPTHNVAEGYRWDIVAQRACQCLKQDCCYDSKNNPVPSWNVKR
ARDIQVTVEQWGYPDLILIVNPYEPIRKVKEKIRRRQGCSGLQRLSFQTPSGERKLLSSRSSLADYGIFC
NTQICLLETTSPEIQVFVKNPDGGSHAYAIDPNSFILGLKHQIEDKQGLLRKQQQLQFRGQVLQDWSSFR
SYGIEDSDTLILSKKKDTDFLFLPS

>XP_036210168.1 2'-5'-oligoadenylate synthase 1 isoform X1 [*Myotis myotis*]
MWPLQAIVARDLEVIPAKDLDRFIEHHLLPDTAFRMQVKQAIDIICSFLKERCFLQDRVRVSKVVKGGSS
GKGTALRDRSDADLVVFLSPLTSFQEQFERRGEFIQETRTQLEACQQQRMFTVRFEVQPKFQNPRALSFT
LKSRSSRAEAEVEFDVLPAYDVLGQVTEPYRPDPDIYVRLIRECERLGKEGEFSPCFTELQRAFLKQRPA
KLKSLIRLVKHWYQKCKEKLGQSLPPQYALELLTVYAWERAGRQTDFITAQGFQTVLELVMNYRQLRIHW
TQYYDSSHPVIGPYLRRQLQKPRPVILDPADPTGNVAGGKPERWEPLAGEARAWMKYPCFRKGDGSPVGS
WNI

>KAF6282567.1 2'-5'-oligoadenylate synthetase 2 [*Myotis myotis*]
MGNWLSWLSGACPKTVYSVPAQKLDEYIQTSLRPTEDCQRQIDEAVDTICAALQEATEPPTVTDVAKGGS
YGRKTVLKGNSDGTLVIFISDLEKFQDQKENQHEILDKIWEQLRACQLERKLTVKMEMQRSNGDLTVRLS
TRWQSVTFDVLPAFDALDLSGKPSPSTYRDLKRALDQTKASPGEFSVCFTKLQEDFFNKHPRKLKDLILL
VKAWHRQCQKLWGDQSLPPSYALELLTVYAWEEGCREEDFDIVQGLRTVLELIEQQEQLCVYWTVNYNFE
DETVRNVMLNQIRSSRPVILDPTDPTNMMSNDETCWRLLKAEAQNWLSSLSLSGSPGPSWNVLPAPIHET
PGHLLDKFIKDFLQPTKKVNIQIKAAVGIISKFLKEICFRHSATKVQKPIKAGSSGKGTALRTGSDADLV
VFVDSLTSFPSQKTERYNVIKEIKHQLEAHQQQVTDQDVEVTFEISKWEAPRVLSFSLKSKHLNGSVDFD
VLPAFNALEKLGPLWKVYTKLIAQYEQHSDTPGGEFSACFTELQRDFVSSRPTKLKNLLRLVKHWYKQCE
RRLKKKGSLPPKYALELLTIYAWEQGSGATSFDTTQGFRTVLELVTRYQQLCVFWTVNYNFENETVRNFL
LKQLQRPRPVILDPADPTGDVGGGNRWCWHHLANEAAEWLSSSLCFKDGTGGPVPSWKVPTVQTPGSCGA
GVGPVINEMFSSRRRGILD

>KAF6282568.1 2'-5'-oligoadenylate synthetase 3 [*Myotis myotis*]
MDVYRTPAAALDGLVASSLQPPAEFVGTVRRALGDLGAALRERGGRPGPGPGPGAAAPAWRVLKIAKGGS

SGRGTALRGGCDSELVVFLDCFKSYEDQRALQTEILKEMRVLVGSWWQKPVPGLSLKFLKQDTPGALQFR
LASTDLENWMDISLVPAFDALGQLSSSVKPKPQVYATLLESGCQKGEHAACFAELRRNFMNTRPAKLKNL
ILLVKHWHRQVCPKAGQNQMPPAYALELLTIFAWEQGCGKEAFSLAQGLRTVLGLIQQYQHLCVFWTTNY
SCEDPAVKKFLQRQLEGPRPVILDPADPTWDVGNGAAWRWDLLAQEAESCYDSPCFLQAAGGAVQPWEGP
GLPRTGRSGVDHPILRDSAQRTPTDSSSVDAGCSRAGDRRPSCPAPGPSVDGITPGTPEGVPGLSQVPAK
ELDRFIQDHLKPSPQFQKQVSKAVDGILGRLRENCVYKASRVGKGGSFGRGTDLRGGCDAELVIFLNCFE
NYKDQGPRRAEILADMRDQLESWWRDPVPGLTLNFPEQTMTKALQFQLMSPALASWVDVSLLPVFDAVGQ
LSAGTKPDPKIYRTLLDSGCQDGEHRACFAELRRSFVNSRPTKLKSLILLVKHWHRQVAARNKGERPARA
SLPPAYALELLTIFAWEQGSGKDRFSTAEGLRTVLGLVQQHRQLCVFWTVNYSSEDPALRAHLLGQLRKP
RPLILDPADPTWNVGLGSWELLAQEAAALETQACFMSADGTPVQPWDVMPTLLYQTPARDLDKFISDFLQ
PNRQFLAQVNKAVDTICSFLRENCFQNSPIKVLKVVKGGSSAKGTALRGRSDADLVVFLSCFGQFTEQGS
TRAEVISEIRAQLEVCKRARQFEVKFNLPKWENPRVLSFSLTSQTMLDQSVDFDVLPAFDALGQLAPGSR
PPPQVYVDLIRSYNTPGEYSSCFTELQRDFVVDRPTKLKSLIRLVKHWYRQCKKMPKGKGSLPPQHGLEL
LTVYAWEQGGQDPQFGMAEGFRTVLELVTQYRQLCVYWTVNYSREDQTVKDFLEQQLRKPRPIILDPADP
TGNLGPNARWDLLAKEAAACMSSLCCIDRDGTPIQPWPVRAAV

>XP_036199939.1 2'-5'-oligoadenylate synthase 1-like [*Myotis myotis*] = OAS5
MELSLTPARNLDKFIEDHLLPDTPFRRTVKKAVNAICSFLKERCFQGASHCVRVSKVVKGGSSGKGTTLR
GRSDADLVVFLSELRSFQEQLDRRGEFIWEIIRQLEAFQRETGVEFEIHKPQREKPRALSFVFRSPGLDE
KVEFDVLPAFDVLGQWGTVPHIHAGYRPDPHIYVRLIHECTRLGKEGEFSPCFTELQRAFLRQRPTKLKS
LIRLVKHWYQLCKQRLGKPLPPQYALELLTVHAWERGCKETYFSTAEGFRTVLELVVHYRQLCVYWTKYY
NFENPVICQYLMTQLEQPRPVILDPADPTGNVAGGGGWQRLAQEAAAWLRYPCVENRDGSPVDSWDTGLP
REREDSWTCAIL

>XP_036209649.1 2'-5'-oligoadenylate synthase-like protein isoform X1 [*Myotis myotis*]
MPGAGCVTTYLPGWVLSHSHGTVRGDGAVRHPCLQAGLLRGSVPAAQPGVEGRGAGGREDRGAVPEGRAP
RGRVWAGPGGAGAEAGEGGLLRERHGARGQRGGAAGGLSELLRRLPGGGRAPRRRAEADPEHAVDLPGPA
GPRARGPGGVPGRPRCSRLHRPDQVDRGAGHRHRRAGLQGPGAFCSPLAAAPRGLCEPGEGLPVPRASLS
FLLRAAERLREAPAHQAEEPPAAGQTLYVRARSPRAALPPLYALELLTIYAWEMGTQENESFRLDEGLTT
VMELLQEYESLCIYWTKYYTFQNPIIEDVVRKQLQRQRPIILDPADPTYNVAEGCRWDIVAQRACQCLKQ
DCCYDSKDNPVPSWNVKRARDIQVTVEQWGCPDWTLMVNPYSSIKKVKEKIQRRQSCEGLQRLSFQTPSG
ERKLLRSRSSLAEFGIFSDTRICLLETIPPEIQVFVRNPEGGSHAYAVDPNSLVLGLKQQIEDKQGLLRK
QQELQFLGQVLQDWSVLGSYGIKDSDTLILSKKKDTHFPFLPS

>XP_036989359.1 2'-5'-oligoadenylate synthase 1 [*Artibeus jamaicensis*]
MELRRTSAKDLDKFIENHLLPDSPFRDRVNEAIHIICSFLKERCFLQGGGRPVRVLKVVKGGSSGKGTAL
RGRSDADLVVFLSSFGSFQEQFDHREEFIQEIRRQLEACRREKMFQVQFEIRKNTAMMQSHNTAVVQSHR
WGNSRVIRKPRSLSFTLSSCSWSCPQEGVEFDVLPAFDVLGQVTKDYRPDPQIYVKLIQECEGLGREGEF
STCFTELQRAFLKQRPAKLKSLIRLVKYWYQECKQELGHPLPPQYALELLTVYAWERGSREADFIMAEGF
RTVLELVLNYRRLCIYWTKYYDTNHSVIGPYLRRQLQKPRPVILDPADPTGNVGADNPPGGWERLAGKAR
DWLTYPCFKKWDGSPVGSWNVQI

>XP_036989379.1 LOW QUALITY PROTEIN: 2'-5'-oligoadenylate synthase 2 [*Artibeus jamaicensis*]
MGNWFSSACPVTVNSVPAQRLGEYVQNFLRPSDDCQRQIDEAMDTICAALEEAEELPTVTDVAKGGSYGR
KTVLRGNSDGTLVIFISDLGQFQDLKRKSQREILNKICKWLKAAQLEQKLAAKMEIKRPWGGGLTIQLST
RWQSVTFDVFPAFDAVGFRNEPCLRTYRELKRSLDMTKASPGEFSVCFTKLQQKFFNHHPRKLKDLILLV
KAWYQQCQKKWKDTSLPPSYALELLTVYAWEQGCGAENFDLAEGIRTVLELIKGPEELCVYWTVNYNFED
MTVRNVILSQIRSPRPVILDPTDPTNNVSKDKTGWQLLKEEAQLWLSSPSLKEPPGPSWNVLPMPLEETP
GHRLDAFIKKFLQPNQTFLNQMSSAVDIICTFLREQCFRDSSTKVQKPVKGGSTSKGTALKTGSDADIVL
FIDSLVGYTSQKNEVSIVVQKIRNQLEAFQRREAREQKFEVKFEISKWNDPKVLSFSLKSKEFSDSVDFD
VLPAYNALGQVHAGVRPHPRVYTDLISLYKRLDLVGGEFSPCFTELQRDFVLSRPTKLKDLIRLVKHWYK
QCARKLKEKGSLPPKYALELLIYAWEQGAGXADFDTAEGFRTVLELVTQYQQLCIFWKVNYNFDNQTVKD
FLLTQIQKTSWRGQAQPRAREPGRPLGAESNPSRQLAPERGPPPCNRVEPAPPTAQKPRASSWTCGTHSS
ATRQAAGL

>XP_036989355.1 2'-5'-oligoadenylate synthase 3 isoform X1 [Artibeus jamaicensis]
MDVYRTPATTLDGLVTSLQLTAEFVGTARRALGTVGAVLRECGGRPGARVAAAPPWRVLKITKGGSFGRG
TALRGGCDSELVVFLNCFKSYEDQRARRREILGEMRVLLESSWQNQVSGLSLEFLKQDMPGVLQFRLAFP
DLDDQMDVSLVPAFDALGQLSSHVKPKPQVYSALLNSGCQRGEHAACFAELRRNFVNTRPPKVKNLILLV
KHWYRQVCPQEGSREMPPAYALELLTIFAWEKGCGKDAFSLAQGLQTVLGLIQQYQHLCVFWTTNYGFED
PVVKKFLLHQLERPRPVILDPADPTWDVGNGAAWAWDLLAQAAGSCCDRFPFLQAVGGAVKPWEVPGLPR
PGRSGLDLPILEDPAREPSRDSSGLSAACPGAGNRRPFRPAPVFSALDLCQIPSWELDRFIMDHLKPDTL
FQRQVSKAIDVILSCLREKCVHKASRVSKGGSFGRGTDLRGGCDAELVIFLNCFENYKDQGPRRAAILDD
MWAQLESWWQDPVPGLTLSFPEQTMTEALQFHLVSPTLKSRMDVSLLPVFDAVGQLSSGTKPDPQIYCTL
LDSGCQEGEHAACFSELRRNFVNTRPAKLKNLILLVKHWYRQVTQVASRNRAGWPACASLPPAYALELLT
IFAWEQGSGEHRFHMANGLRTVLGLVQQHQQLRVFWTVNYSFEDPALRMHLLSQLQKPRPLILDPADPTW
NVGRGSWELLAQEAAALESQACLMSREGIPVQPWDVTPTLLHQTPARDLDKFISDVLQPDRQFLGQVHKA
VDTICSFLRENCFKNLPIKVLKVVKGGSSAKGTALRGHSDADLVVFLSCFHQFTDQGSRRAEIISEIRAQ
LERCKQEQQFEVKFEISRWENPRVLSFSLASQTMPDQSVDFDVLPAFDALGQLGSGSRPHPRVYADLIRS
CSNAGEYSCCFTELQRDFIVSRPTKLKSLIRLVKHWYRQCTKKPKGKGSLPPQHGLELLTVYAWEQGGRD
PRFNMAEGFRTVLELVTRYRQLRVYWTVNYNCEDETIRDFLRTQLQKARPIILDPADPTGILGHSARWDL
LAEEAAAYMSAPCCMDRDGTPILPWPVKAAV

>XP_037010350.1 2'-5'-oligoadenylate synthase 1-like [*Artibeus jamaicensis*] = OAS5
MCQEANTRSQLLPSQERKPKPVQTGQQKTQRLRVSRRQLCGHCSPVPCQSVKMELSEIPARCLDKFIEDH
LLPDTHFRMQVKEAINVICSFLKERCFRGASHPVRVSKVVKGGSSGKGTTLRGRSDADLVVFLSNLGSFQ
EQFDGRAEFIREIKKQLTACQSERGVQFKIRDGGWWNPRSLSFVLRSFWVEGVEFDVLPAFDALGQVPKG
YAHDSLVPEDYRPDPQIYIELIQECKKLGKEGEFSTCFTELQRAFLKQRPTKLKSLIRLVKHWYQTCKEN

LGKPLPPQYALELLTVYAWEQGNKGTVFSTAQGFRTVLELVMNYQKLLIYWTKYYSFENPVIGQYLMMQL
KKPRPVILDPADPTGNVAGGDPRSWPRLAQEAKAWLSYQCFKNWDGSPVSPWYIQLPAESEDGWTCAIL

>XP_037012124.1 2'-5'-oligoadenylate synthase-like protein [*Artibeus jamaicensis*]
MALSPELYGIPASRLDSFVAQWLQPNREWKEEVLDAVRTVEQFLRAEAFQGEDGLDQEVQVLKVLKVGSF
GNGTVLRSSAEVELVVFLSCFHSFQQEAKHHQAVLRMIRKKLWTCQDLLALGLEVLGVLQGVPDALTFTI
QTRWTAELVTVTIVPAYRALGPSVSNSQPHPEVYVSLIKAGGYPGNFSPSFCELQRNFVKHRPTKLKSLL
RLVKHWYLQYVKAKCPRAMLPPLYALELLTIYAWEMGTLEDENFSLDEGLVTVMELLQKYQSLCIYWTRH
YAFENPIIEDCVRKQFQRERPIILDPADPTHNVAEGYRWDIVAQRANQCLKQDCCYDNEETPVPSWNVKT
ARNITVTVEQWGYPDLILIVNPHETIKNVKKKIRRRQGYSGQQRLSFQQPSGERQLLNGCSSLADFGIFS
DTCISLLETMASEILVFVKNPGGGSHAYAIDPKSFVLSLKQQIEDKEGILRNQQQLEFQGQVLQDWLAFE
GYGIKDSDTLILSKKKVRRVPFVLS

>XP_010597281.1 2'-5'-oligoadenylate synthase-like protein isoform X1 [*Loxodonta africana*]
MALAQELYDTPASRLDSFVAQWLQPSREWKEEVLGVVRTVERFLREEHFQGDRGLDQEVRVLKVVKAGSF
GNGTVLRGTAEVELVVFLSCFRSFQEEAKYHQAVLRLIQKKLWRCQDLLALGLEDLWVAQGAPGALVFTI
QTSGIEELITITIVPAYRALGPLAPNFQPPPDVYVSLIKAHGYPGNFSPSFSELQRNFVKHQPTKLKNLL
RLVKHWYLQYVKAKCPRANLPPFYALELLTIYAWEMGTQKDENFRLDEGLTTVMELLQAYEFVCIYWTKY
YTFQNLVIENFVRKQLKKDRPIILDPADPTRNVAEGYRWDIVAQRACQCLKQDCCYDNQEFPVTSWNVKR
ARDIQVTVEQRNYFDLILWVDPYEPIKKIREKIQQNRGYSGLQRLSFQEPGGERQLLSSHCSLAYYGVFS
NTRIYLLETFSPEIQVFVKNQDGRSHAYAIDPNNCILRLKEQIEDRLGLLRRQQQLEFRGQVLQDWLDFG
CYGIQDSDTLILSKKKASEAPFLHS

>XP_023415269.1 2'-5'-oligoadenylate synthase-like protein 2 [*Loxodonta africana*]
MEHLQNLYETPADRLDAFVSHSLQPQGDWKEEVQDAWQRIERFLRDQCFRDELVLDQEVRVLKVVKGGSS
GKGTTLNYSSDVDMVLFLSCFSSFQNLAQLRKDIIHFIQEKLDHRSRSLAYNISLVQPRERARAPRSLSF
RVQSRKSSEAIWVDVLPAFNALGSFCPDTKPSPEVYEDLITSAGQPGEFSSSFTELQKHFVKSCPTKLKS
LLRLVKHWYLQHLKPKYRKVQLPSKYALELLTIYAWEMGTDESENFDMDEGFVAVMKLLRDHEDICIYWT
KYYDFQNEIVRNYLKQKLKECRPIILDPADPTNNLGKGKRWDLVAKEAVHCLRQPCCDIEDPGQGWHIQP
ARDVQVTVKKSGEEAQTLSVNPYSPIWKMKKEIKNMFFLNGHQRLSFQEPGGKRQPLSSQKTLADYGIFS
KVGIRVLETFPPEIQIFVKEPSGQNRPYAVRPDGLIWHLKTNIEAAGGPCLEDQILKFQGRNLRNHLSLR
DLQIRTVTHHPIRRGCQVPGKRHRFLLGPSQVRLS

>XP_012629210.1 2'-5'-oligoadenylate synthase 1-like [*Microcebus murinus*] = OAS4
MWALKFYNCEHCGSDFVSHTALKYHLRLKHLGKVHCEECGQEVLREKLEDHSKSVHDRSIVETHNLNWEN
LEDRSIKHVATKISKTHRMCSEYFGTVHSQEIPEIQEHHFIANKRAQMSTGFSPHNMLECKSPQELERFA
DENIRPASGPLSAACVAEIGAFTELIQGCFPAPASRVIQGGSYTKGTDTQGCSEVDIVLFSDVFANVNNC
KKQLREGLDVLRKNLKQTSRGNRIIMKKRTSLSLRFNFLCIESLHSHSFEIMAYYDVLGPSPSKDLKLHL
YRKLYLCNDIEEAQLCALALLPYQVDFVKASIVRVKELIRLMTHWFKTSFAKPTEKNKFRRLPSSYAVEL
LSIYIWELAGKPLLFSLVQGMRAVLKLLVRYAEIDIVWHRHYHSKFPIFVKVKQKHTRPFILDPVNPTVN
VCDTCNAWDEVARVARCSLCQPLFSGVRAKPPWLFTDDWY

>XP_039400985.1 2'-5'-oligoadenylate synthase 1-like isoform X1 [*Mauremys reevesii*] = OAS4
MDKGDSEGHCASPCKNPGLASCAESYYEITRKSQPEMKVWAEGHGITAGCCPYSVGAQERERERMGSLKF
YNCEQCGNDFVSRTALRYHFRAKHLGKVHCEKCGREVLRETLQDHEAANHERMTAERQGCFWDMPPKREA
KFVAIEMSTSCRMCPRKFGTIHSREHHEKQDHQFTASKRAQMATGFSPDNILDYKNPTELKRFVEEKLRP
VPGPASIACATETEAIINLIKECFPLPIARLIKGGSYIKGTDTQGWSDVDIVLFSEAFENLEDCKKKLPE
VIDDLGKRLKKSSWASRIMMEKRTQSSLRFHFKCYKNHHGHSFDIMPCYDMLGPAPSTGFKQSFYHKIYL
CNDTDEIQLYSMSLLQYQVEFIKASTMKVKDLIRLVKHWFRTSFAKPTAQNKFRRLPSSYAIELITISIW
QLAGKPVFFSLIQGMRAILMLLVRYPEMCIVWHKHYSPNSMIFKKAFQKQTRPFILDPANPTLNVCENSN
AWDEVAHVARRSLLKPLFNGVQAKEPWLFTNDW

Figure S1. Amino acid sequences of OAS family proteins encoded by genes which have been investigated in this study. Amino acid sequences of OAS proteins are shown in FASTA format. Annotation as OAS4 or OAS5 is indicated at the end of the name. Note that some proteins are designated "low-quality" in GenBank because the nucleotide sequences of the corresponding genes contain at least one frame shift or premature stop, which may be attributed to genome sequencing errors or genuine mutations. The position of ambiguous sites is marked by X (red font) in the sequence. OAS1 and OAS2 of the Chinese pangolin (*Manis pentadactyla*) were predicted as described in Figure S3. In addition to proteins listed in Table S1, this list contains sequences of proteins which were used for inferring the evolutionary origin or loss of OAS family genes during the evolution of vertebrates. The latter proteins are from the following species: African savannah elephant (*Loxodonta africana*, order: Proboscidea, superorder: Afrotheria); gray mouse lemur (*Microcebus murinus*, order: Primates, superorder: Euarchontoglires), Reeves' turtle (*Mauremys reevesii*, order: Testudines, class: Reptilia).

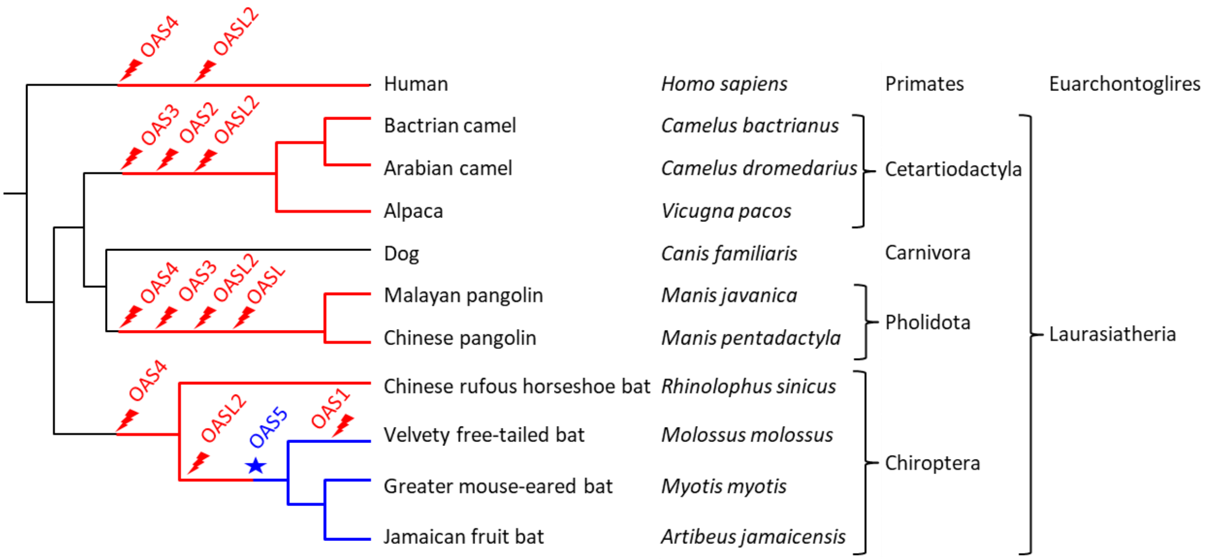

Figure S2. Phylogenetic tree of species investigated in this study and mapping of gene loss and gain events. The tree shows the relationship of species. Higher taxonomic ranks are shown on the right. Gene loss (flash symbol, red) and gain (star, blue) are mapped onto this tree on the basis of gene absence or presence in the extant species. Red lines indicate lineages in which at least one member of the OAS gene family was missing. Blue lines indicate presence of *OAS5* in Yangochiroptera. The chronological order of gene loss events between nodes of the tree is not known.

A

Manis pentadactyla OAS1

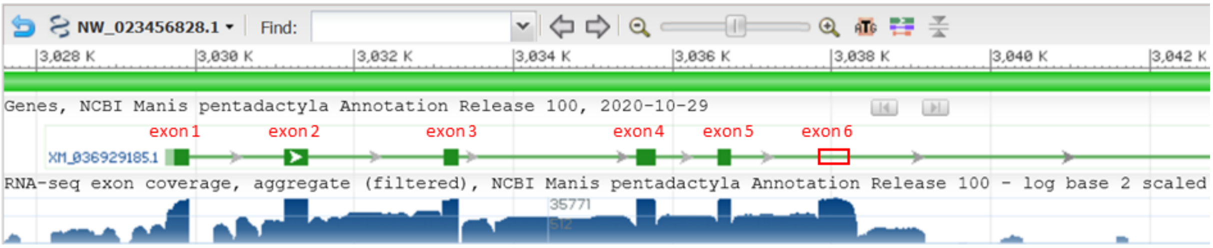

B

OAS1 exon 6 (coding sequence)

```
ccatgtgctttaccctttcagcccagagacaacctctgctccataccctgagtatggaca
                      P  R  D  N  L  C  S  I  P  *
```

C

Manis pentadactyla OAS2

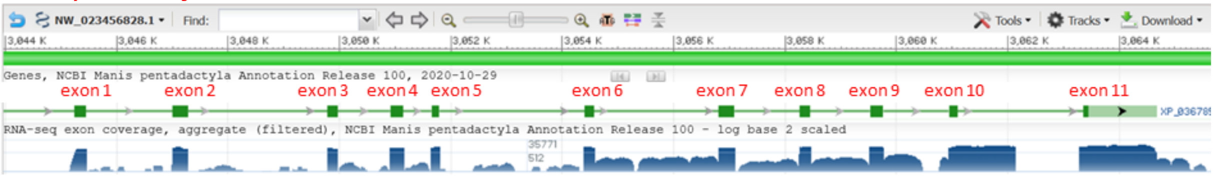

D

OAS2 exon 1 (coding sequence)

```
ctctgcctccctctctgccattagctgtcacctcagcagccagcagaagccatgggcaac
                                                    M  G  N
tgggaatcccatctgtattcagtgcctcctcagaaactgggtgagtttgtccagaactcc
 W  E  S  H  L  Y  S  V  P  P  Q  K  L  G  E  F  V  Q  N  S
ctgagaccctttgaagattgtcagaaaaagatcgacgagaccgtgaacaccatctgcgct
 L  R  P  F  E  D  C  Q  K  K  I  D  E  T  V  N  T  I  C  A
gtcctgcaggaagctgagcagttccccctggtcataagtgtggctaaagtgagtacaggg
 V  L  Q  E  A  E  Q  F  P  L  V  I  S  V  A  K
```

Figure S3. Prediction of exons for *OAS1* and *OAS2* genes of the Chinese pangolin (*Manis pentadactyla*). GenBank Gene views of LOC118934083 2'-5'-oligoadenylate synthase 2-like [*Manis pentadactyla* (Chinese pangolin)], Gene ID: 118934083, updated on 29-May-2021. The LOC118934083 gene prediction in GenBank erroneously links the OAS1 and OAS2 genes. Positions of exon 6 of *OAS1* (A) and exon 1 of *OAS2* (C) are indicated in the Gene views. Nucleotide sequences of exon 6 of *OAS1* (B) and exon 1 of *OAS2* (D) contain coding sequences (red fonts) and adjacent sequences. The amino acid sequence is shown below the coding sequences. Splice donor and acceptor sites are underlined.

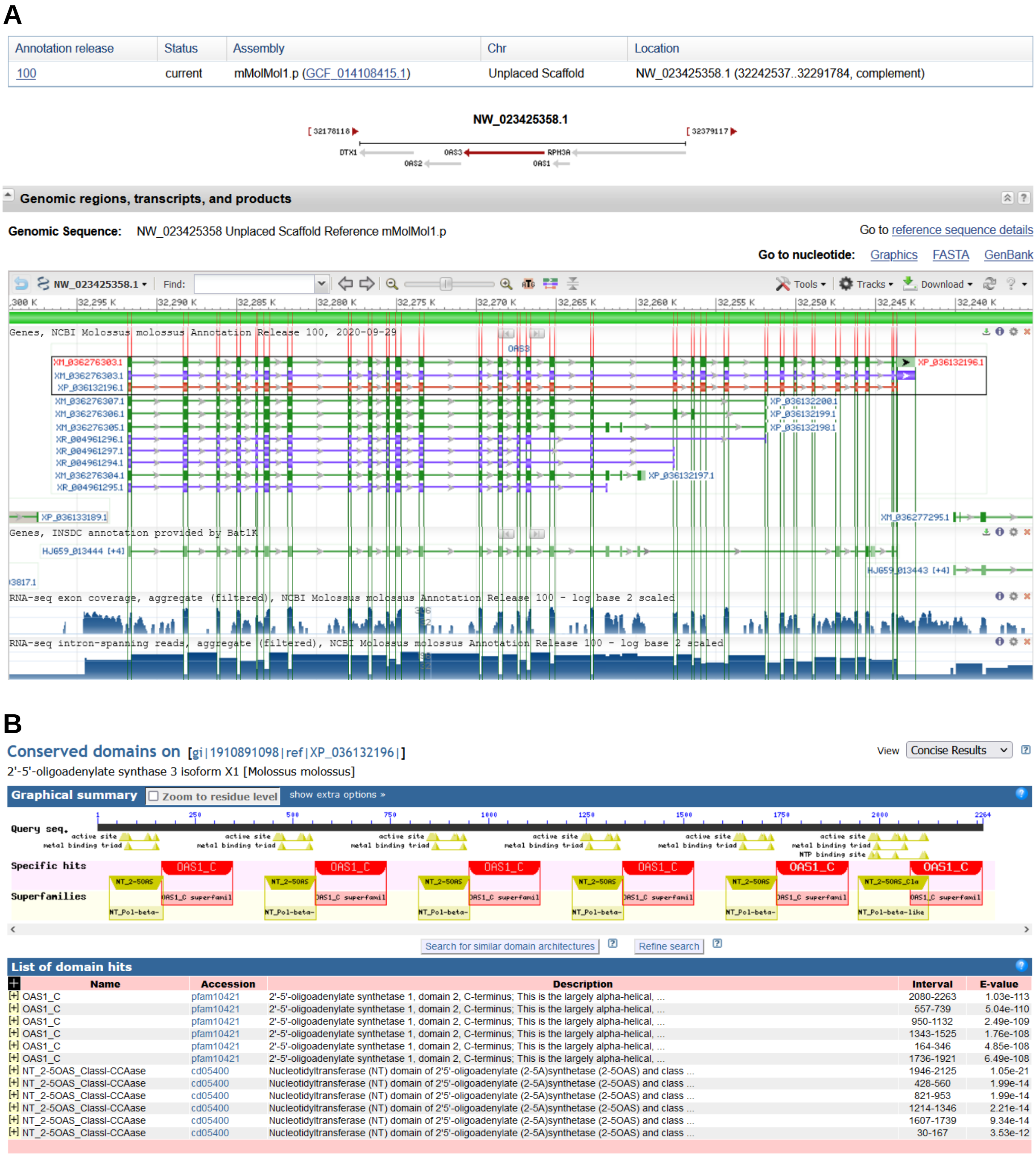

Name	Accession	Description	Interval	E-value
OAS1_C	pfam10421	2'-5'-oligoadenylate synthetase 1, domain 2, C-terminus; This is the largely alpha-helical, ...	2080-2263	1.03e-113
OAS1_C	pfam10421	2'-5'-oligoadenylate synthetase 1, domain 2, C-terminus; This is the largely alpha-helical, ...	557-739	5.04e-110
OAS1_C	pfam10421	2'-5'-oligoadenylate synthetase 1, domain 2, C-terminus; This is the largely alpha-helical, ...	950-1132	2.49e-109
OAS1_C	pfam10421	2'-5'-oligoadenylate synthetase 1, domain 2, C-terminus; This is the largely alpha-helical, ...	1343-1525	1.76e-108
OAS1_C	pfam10421	2'-5'-oligoadenylate synthetase 1, domain 2, C-terminus; This is the largely alpha-helical, ...	164-346	4.85e-108
OAS1_C	pfam10421	2'-5'-oligoadenylate synthetase 1, domain 2, C-terminus; This is the largely alpha-helical, ...	1736-1921	6.49e-108
NT_2-5OAS_ClassI-CCAase	cd05400	Nucleotidyltransferase (NT) domain of 2'5'-oligoadenylate (2-5A)synthetase (2-5OAS) and class ...	1946-2125	1.05e-21
NT_2-5OAS_ClassI-CCAase	cd05400	Nucleotidyltransferase (NT) domain of 2'5'-oligoadenylate (2-5A)synthetase (2-5OAS) and class ...	428-560	1.99e-14
NT_2-5OAS_ClassI-CCAase	cd05400	Nucleotidyltransferase (NT) domain of 2'5'-oligoadenylate (2-5A)synthetase (2-5OAS) and class ...	821-953	1.99e-14
NT_2-5OAS_ClassI-CCAase	cd05400	Nucleotidyltransferase (NT) domain of 2'5'-oligoadenylate (2-5A)synthetase (2-5OAS) and class ...	1214-1346	2.21e-14
NT_2-5OAS_ClassI-CCAase	cd05400	Nucleotidyltransferase (NT) domain of 2'5'-oligoadenylate (2-5A)synthetase (2-5OAS) and class ...	1607-1739	9.34e-14
NT_2-5OAS_ClassI-CCAase	cd05400	Nucleotidyltransferase (NT) domain of 2'5'-oligoadenylate (2-5A)synthetase (2-5OAS) and class ...	30-167	3.53e-12

Figure S4. Predicted structure of OAS3 in Pallas's mastiff bat (*Molossus molossus*). (A) GenBank Gene view of *OAS3 2′-5′-oligoadenylate synthetase 3* [*Molossus molossus* (Pallas's mastiff bat)], Gene ID: 118639793. (B) Domain prediction of the OAS3 protein of *Molossus molossus*. The National Center for Biotechnology Information (NCBI) and the National Library of Medicine (NLM) are acknowledged for tools and information provided in the public domain.

```
                  1                                                                                                  100
Human-Hsa_OAS1   ----------------------------------------------------MMDLRNTPAKSLDKFIEDYLLPDTCFRMQINHAIDIICGFLKERCF-R
  Dog-Cfa_OAS1   ----------------------------------------------------MPELKDTPAKDLDRFIENYLLPDTQFRRQVKEAIHIISTFLKERCF-Q
  Bat-Rsi_OAS1   -----------------------------------------------------MELRNTPAKNLDKFIQDHLLPDKHFRSQVKQAIDSICSFLKEKCF-Q
  Bat-Mmy_OAS1   --------------------------------------------MWPLQAIVARDLEVIPAKDLDRFIEHHLLPDTAFRMQVKQAIDIICSFLKERCFLQ
  Bat-Aja_OAS1   -----------------------------------------------------MELRRTSAKDLDKFIENHLLPDSPFRDRVNEAIHIICSFLKERCFLQ
  Bat-MMo_OAS5   -----------------------------------------------------MQLRDTQARFLDKFIEENLLPDTPFRLQVRQAIHHICGFLKEECF-Q
  Bat-Mmy_OAS5   -----------------------------------------------------MELSLTPARNLDKFIEDHLLPDTPFRRTVKKAVNAICSFLKERCF-Q
  Bat-Aja_OAS5   MCQEANTRSQLLPSQERKPKPVQTGQQKTQRLRVSRRQLCGHCSPVPCQS-VKMELSEIPARCLDKFIEDHLLPDTHFRMQVKEAINVICSFLKERCF-R

                 101                                                                                                 200
Human-Hsa_OAS1   GSSYPVCVSKVVKGGSSGKGTTLRGRSDADLVVFLSPLTTFQDQLNRRGEFIQEIRRQLEACQRERAFSVKFEVQAPR---------------------W
  Dog-Cfa_OAS1   GAAHPVRVSKVVKGGSSGKGTTLRGRSDADLVVFLNNLKSFQEQLEKRGQFIWEIKRQLEACQREETFEVYFEVQSLQ---------------------W
  Bat-Rsi_OAS1   HASHSVRVSKVVKGGSSGKGTTLRGRSDADLVVFLSPLRSFQEQFDRRGEFIQEIRKQLEACQREETFAVEFEVQNHR---------------------W
  Bat-Mmy_OAS1   DR---VRVSKVVKGGSSGKGTALRDRSDADLVVFLSPLTSFQEQFERRGEFIQETRTQLEACQQQRMFTVRFEVQPK----------------------F
  Bat-Aja_OAS1   GGGRPVRVLKVVKGGSSGKGTALRGRSDADLVVFLSSFGSFQEQFDHREEFIQEIRRQLEACRREKMFQVQFEIRKNTAMMQSHNTAVVQSHRWGNSRVI
  Bat-MMo_OAS5   GAPHRVRVSKVVKGGSSGKGTTLRGRSDADLVVFLSPLTSFQEQFDRRGEFIQEIKKQLQAFQRET--GENFEIRDSG---------------------W
  Bat-Mmy_OAS5   GASHCVRVSKVVKGGSSGKGTTLRGRSDADLVVFLSELRSFQEQLDRRGEFIWEIIRQLEAFQRET--GVEFEIHKPQ---------------------R
  Bat-Aja_OAS5   GASHPVRVSKVVKGGSSGKGTTLRGRSDADLVVFLSNLGSFQEQFDGRAEFIREIKKQLTACQSER--GVQFKIRDGG---------------------W

                 201                                                                                                 300
Human-Hsa_OAS1   GNPRALSFVLSSLQLG---EGVEFDVLPAFDALG----------QLTGGYKPNPQIYVKLIEECTDLQKEGEFSTCFTELQRDFLKQRPTKLKSLIRLVK
  Dog-Cfa_OAS1   EKPRALSFVLKSPQLG---EGVEFDVLPAFDVLG----------QWTNNHRPNPEVYIKLIQECENRGTEGEFSTCFTELQRDFLRQRPTKVKSLIRLVK
  Bat-Rsi_OAS1   TNPRALGFVLKSPTIR---DGVEFDVLPAFDVLAD---------QVTDNYIPDPQIYVDLIQACEHLKKEGEFSTCFTELQRAFLRQRPTKLKSLIRLVK
  Bat-Mmy_OAS1   QNPRALSFTLKSRSSRAEAE-VEFDVLPAYDVLG----------QVTEPYRPDPDIYVRLIRECERLGKEGEFSPCFTELQRAFLKQRPAKLKSLIRLVK
  Bat-Aja_OAS1   RKPRSLSFTLSSCSWSCPQEGVEFDVLPAFDVLG----------QVTKDYRPDPQIYVKLIQECEGLGREGEFSTCFTELQRAFLKQRPAKLKSLIRLVK
  Bat-MMo_OAS5   QNPRVLSFVFSSPMLN---DSVEFDVLPAFDVLGQ------------LTGPPDPKIYVQLIDECTRLGKEGEFSPCFTELQRDFLKQRPTKLKSLIRLVK
  Bat-Mmy_OAS5   EKPRALSFVFRSPGLD---EKVEFDVLPAFDVLGQ----WGTVPHIHAGYRPDPHIYVRLIHECTRLGKEGEFSPCFTELQRAFLRQRPTKLKSLIRLVK
  Bat-Aja_OAS5   WNPRSLSFVLRSFWV----EGVEFDVLPAFDALGQVPKGYAHDSLVPEDYRPDPQIYIELIQECKKLGKEGEFSTCFTELQRAFLKQRPTKLKSLIRLVK

                 301                                                                                                 400
Human-Hsa_OAS1   HWYQNCKKKLGK-LPPQYALELLTVYAWERGSMKTHFNTAQGFRTVLELVINYQQLCIYWTKYYDFKNPIIEKYLRRQLTKPRPVILDPADPTGNL-GGG
  Dog-Cfa_OAS1   HWYQKCKQKLGKPLPPQYALELLTVYAWERGNHQTEFITAQGFQTVLKLVLNYQQLCIHWTKYYNFETPIIKQYLMRQLAKPRPVILDPADPTGNV-AGR
  Bat-Rsi_OAS1   HWYQTFKEKCGESLPPQYALELLTVYAWERGGEKPEFNTAQGFKTVLELVRGYQQLWIYWPKYYNFKNPVICHYLMKQLEKPRPVILDPADPTGNV-GGG
  Bat-Mmy_OAS1   HWYQKCKEKLGQSLPPQYALELLTVYAWERAGRQTDFITAQGFQTVLELVMNYRQLRIHWTQYYDSSHPVIGPYLRRQLQKPRPVILDPADPTGNV-AGG
  Bat-Aja_OAS1   YWYQECKQELGHPLPPQYALELLTVYAWERGSREADFIMAEGFRTVLELVLNYRRLCIYWTKYYDTNHSVIGPYLRRQLQKPRPVILDPADPTGNVGADN
  Bat-MMo_OAS5   HWYQVCKEKLGKPLPPQYALELLTVYAWERGSNRSEFSTAQGFRTVLELVMNYRMLCIYWSKYYNFENPVIGAYLRKQLQKPRPVILDPADPTGNV-AGG
  Bat-Mmy_OAS5   HWYQLCKQRLGKPLPPQYALELLTVHAWERGCKETYFSTAEGFRTVLELVVHYRQLCVYWTKYYNFENPVICQYLMTQLEQPRPVILDPADPTGNV-AGG
  Bat-Aja_OAS5   HWYQTCKENLGKPLPPQYALELLTVYAWEQGNKGTVFSTAQGFRTVLELVMNYQKLLIYWTKYYSFENPVIGQYLMMQLKKPRPVILDPADPTGNV-AGG

                 401                                                                                     489
Human-Hsa_OAS1   DPKGWRQLAQEAEAWLNYPCFKNWDGSPVSSWILLAESNSADDETDDPRRYQKYGYIGTHEYPHFSHRPSTLQAASTPQAEEDWTCTIL
  Dog-Cfa_OAS1   DTYGWQRLAQEARVWLSYPCFKKRDGSPVGSWDVLLEEDYEDNWITCEHRTYSYHDYGWR--PVSSGSPNTGMTQSIPQQEENWMCTIL
  Bat-Rsi_OAS1   DRRRWQRLAQEAETWLSYPCFKNHDGCPVDSWDIQF-----------------------------------------------------
  Bat-Mmy_OAS1   KPERWEPLAGEARAWMKYPCFRKGDGSPVGSWNI-------------------------------------------------------
  Bat-Aja_OAS1   PPGGWERLAGKARDWLTYPCFKKWDGSPVGSWNVQI-----------------------------------------------------
  Bat-MMo_OAS5   HPQSWPRLAQEALAWLSYPCFKNWDGSPVGSWNI-------------------------------------------------------
  Bat-Mmy_OAS5   G--GWQRLAQEAAAWLRYPCVENRDGSPVDSWDTGLPR----------------------------------------EREDSWTCAIL
  Bat-Aja_OAS5   DPRSWPRLAQEAKAWLSYQCFKNWDGSPVSPWYIQLPA----------------------------------------ESEDGWTCAIL
```

Figure S5. Amino acid sequence alignment of OAS1 and OAS5 proteins. Amino acid sequences were aligned with the Multalin program. Red fonts, residues conserved in all sequences; blue fonts, residues conserved in at least 50% of sequences. The italicized segment of the sequence Bat-Aja_OAS5 is likely to correspond to an erroneous extension of the coding sequence in the protein prediction of GenBank. The accession numbers of the sequences are shown in Figure S1. Species: Hsa, *Homo sapiens*; Cfa, *Canis familiaris*; Rsi, *Rhinolophus sinicus*; Mmo, *Molossus molossus*; Mmy, *Myotis myotis*; Aja, *Artibeus jamaicensis*.
